# Regulatory network of circRNA–miRNA–mRNA contributes to the histological classification and disease progression in gastric cancer

**DOI:** 10.1186/s12967-018-1582-8

**Published:** 2018-08-02

**Authors:** Jia Cheng, Huiqin Zhuo, Mao Xu, Linpei Wang, Hao Xu, Jigui Peng, Jingjing Hou, Lingyun Lin, Jianchun Cai

**Affiliations:** 1 Department of Gastrointestinal Surgery, Zhongshan Hospital, Xiamen University, Xiamen, 361004 Fujian China; 2 Institute of Gastrointestinal Oncology, Medical College of Xiamen University, Xiamen, 361004 Fujian China; 3 Xiamen Municipal Key Laboratory of Gastrointestinal Oncology, Xiamen, 361004 Fujian China; 4 Union Hospital, Fujian Medical University, Fuzhou, 350001 Fujian China

**Keywords:** Stomach neoplasm, circRNA, Ming’s classification, miR-124, miR-29b, ROC curve

## Abstract

**Background:**

Little has been known about the role of non-coding RNA regulatory network in the patterns of growth and invasiveness of gastric cancer (GC) development.

**Methods:**

MicroRNAs (miRNAs) microarray was used to screen differential miRNA expression profiles in Ming’s classification. The significant differential expressions of representative miRNAs and their interacting circular RNA (circRNA) were confirmed in GC cell line and 63 pairs of GC samples. Then, a circRNA/miRNA network was constructed by bioinformatics approaches to identify molecular pathways. Finally, we explored the clinical value of the common targets in the pathway by using receiver operating characteristic curve and survival analysis.

**Results:**

Significantly differential expressed miRNAs were found in two pathological types of GC. Both of miR-124 and miR-29b were consistently down-regulated in GC. CircHIPK3 could play a negative regulatory role on miR-124/miR-29b expression and associated with T stage and Ming’s classification in GC. The bioinformatics analyses showed that targets expression of circHIPK3-miR-124/miR-29b axes in cancer-related pathways was able to predict the status of GC and associated with individual survival time.

**Conclusions:**

The targets of circHIPK3-miR-124/miR-29b axes involved in the progression of GC. CircHIPK3 could take part in the proliferation process of GC cell and may be potential biomarker in histological classification of GC.

**Electronic supplementary material:**

The online version of this article (10.1186/s12967-018-1582-8) contains supplementary material, which is available to authorized users.

## Background

Gastric cancer (GC) is an important public health problem in most parts of the world [1–3]. Although the overall morbidity and mortality of GC is decreasing in many countries, it is still the fourth most common cancer and the second leading cause of cancer death worldwide [2]. At present, environmental pathogenic factors and individual genetic background were thought to result in the gastric carcinogenesis. Genetic risk was found to affect around 10% of cases with GC in familial clustering study [2]. Mutations of some risk genes have also been identified as genetic basis of GC. However, the exact molecular mechanism and the risk gene regulatory network of GC are far from the clear.

GC tissue is composed of cancer cells and various types of stromal cells including endothelial cells and fibroblasts. Cell proliferation and invasion are the main pathological features of GC, which drive malignant tumor cells transfer from a primary site to other areas [4]. Based on patterns of growth and invasiveness, GC was concisely classified into expanding and infiltrative types according to Ming’s classification [5–11]. Expanding carcinoma showed an expansionary growth manner and resulted in the formation of discrete tumor nodules, whereas in infiltrative carcinoma tumor cells invaded individually [5, 8, 10]. These two types of carcinoma appeared to be different in their histogenetic origins and provided a simple basis for evaluation of various clinicopathological aspects of GC. The infiltrative type of GC has a worse prognosis than expanding type [8, 10]. It is interesting to explore the molecular basis in this classification and helpful to develop new treatment strategies for overcoming tumor invasion and metastasis.

MicroRNA (miRNA) play critical biological roles in human carcinogenesis through various molecular mechanisms. Using miRNA-profiling-based screening assay can distinguish types and stage of cancers, and some specific miRNAs may be associated with certain histological subtypes of cancer [12–14]. Circular RNA (circRNA), with the remarkable characteristic of non-canonical splicing without a free 3′ or 5′ end, is widely expressed in human cells and show high tissue-specific expression pattern [15–18]. Several circRNAs can regulate gene expression at post-transcriptional level by inhibiting miRNA activity, some of which promote cell proliferation and serve as an independent biomarker of GC [15, 19–23]. Recently, circRNA 0000284 (circHIPK3) that produced from the homeodomain-interacting protein kinase-3 (HIPK3) gene was found could sponge multiple GC related miRNAs [24, 25]. However, little was known about its role of regulatory network in GC. The main challenge of exploring the impact of regulatory networks to histological classification is the establishment of cell line that origin from specific pathological type tissue of GC. To address this problem, the new GC cell lines of Ming’s classification XGC-1 and XGC-2 were established and characterized in our laboratory as previous report [26]. In this study, our results suggested that the dysregulaton of circRNA–miRNA network might be involved in differential patterns of growth and invasiveness of GC and provided new understanding of the biological role of circRNA–miRNA–mRNA regulatory network in GC development and clinical progression.

## Methods

### Clinical specimens

The snap-frozen GC tissues and matched normal gastric epithelial tissues were recruited from the patients receiving operation in Zhongshan Hospital, Xiamen University. All surgical specimens were confirmed by pathological examination. Tumors were staged according to the tumor-node metastasis (TNM) staging system (7th ed.). Histological grade was on the basis of the National Comprehensive Cancer Network (NCCN) Clinical Practice Guideline of Oncology (V.1.2012). A total of 63 pairs of clinical samples were included, 28 infiltrative type GC samples and 35 expanding type GC samples. No chemotherapy or other antitumor treatments were received at the time when the specimens were obtained.

### Cell culture

The XGC-1 cell line originated from infiltrative type GC and the XGC-2 cell line was established from expanding type GC. The human gastric epithelial cell line GES-1 was obtained from the Cancer Institute and Hospital of the Chinese Academy of Medical Sciences (Beijing, China). Two GC cell lines, MGC-803 and BGC-823, were purchased from the Shanghai Institutes for Biological Sciences, Chinese Academy of Sciences (Shanghai, China). All the cells were cultured in RPMI1640 medium (HyClone, Logan, Utah, USA) with 10% fetal calf serum and incubated at 37 °C in a humidifed atmosphere with 5% CO_2_.

### RNA extraction and microarray analysis

The RNA extraction was performed as previous report [27]. Three paired infiltrative type GC samples and three paired expanding type GC samples were selected to analyze miRNA expression profile between the two types according to Ming’s classification. Total RNA of the paired samples were harvested using TRIzol and an RNeasy mini kit (Qiagen, Germany) according to the protocol of manufacturer. Each group of the RNA samples were labeled and mixed pair-wise using the miRCURY™Hy3™/Hy5™ Power labeling kit and then hybridized on the miRCURY™ LNA Array (Version 14.0, Exiqon, Denmark) which contains 1384 capture probes targeting human miRNAs. Scanning was performed with an Axon GenePix 4000B microarray scanner. The image raw intensity was read using GenePix pro version 6.0 software (Molecular Devices, Sunnyvale, CA, USA).

### Quantitative real-time PCR and bioinformatic analysis

All real-time quantitative reverse transcription polymerase chain reaction (qRT-PCR) process were performed as previous report [4, 28]. The siRNA and specific primers of miRNAs were synthesized by Ribobio (Guangzhou, China) that listed in Additional file 1: Table S1. The investigation of miRNA pathway was carried out based on the instructions of DIANA-miRPath, KEGG and DAVID Database as previous report [28–30]. The graph of the circHIPK3–miRNA–mRNA network in cancer pathway was drawn with Cytoscape (version 3.4.0).

### Cell transfection and proliferation assay

To investigate the biological effect of circHIPK3 in GC cells, circHIPK3 over-expression vector was constructed by using the pHB-circBasic™ circular RNA cloning kit (Hanbio, Shanghai, China). The specially designed front cir-signal and back cir-signal were synthesized and added to the downstream of the CMV promoter in the pHB-circBasic™ vector. In brief, the cDNA encoding liner form of HIPK3 transcript in GES-1 cells was amplified using primers 5-GTATGGCCTCACAAGTCTTG-3 and 5-CTGTAGTACCGAGATTGTAGATATG-3, and then the PCR product was purified by using Gel Extraction Kit (Omega Bio-tek, Doraville, GA, USA). Firstly, we amplified the purified PCR product using the circular primers 5′-CGTACTAATGACTTTTTTTTTATACTTCAGGTATGGCCTCACAAGTCTTG-3′ and 5′-CCTAATTCTTTTCCTTGCTTCTTACCTGTAGTACCGAGATTGTAGATATG-3′. Subsequently, this PCR fragment was purified again and inserted into the site between the front cir-signal frame and back cir-signal frame in the pHB-circBasic™ vector. The result of this fusion vector construction was verified by Sanger sequencing. Finally, GC cells were transfected with circHIPK3 plasmids using the Turbofect transfection reagent (Thermo, Waltham, MA, USA). The siRNA was transfected into cells using ribo FECT ™ CP Transfection Kit (Ribobio, Guangzhou, China). The proliferation of GC cell, MGC-803 and BGC-823, were tested by Cell Counting Kit-8 (CCK-8) assay (Dojindo, Kumamoto, Japan) and performed as previous report [24].

### ONCOMINE and ROC curve analysis

ONCOMINE gene expression array datasets was used to explore gene expression profiles in human cancer. In this study, we analyzed the datasets of Cui [31], which recruited 80 paired GC cases with largest sample size in ONCOMINE, to indentify the transcription levels of COL1A1, COL4A1 and CDK6 between normal gastric tissues and GC tissues. Receiver operating characteristic (ROC) curve was constructed by calculating the sensitivity and specificity of target expression level of circHIPK3–miRNA network in a logistic regression model at different cutoff points for differentiating GC tissues from normal gastric tissues. The status of GC pathological diagnosis is used as the standard of truth.

### Survival analysis

Overall survival (OS) and first progression (FP) curves were calculated with the Kaplan–Meier method to evaluate the prognostic value of COL1A1, COL4A1 and CDK6 mRNA expression in GC. A total of 876 GC patients were recruited from the Kaplan–Meier Plotter online database. Subjects were split into two groups by median expression (high vs. low expression) and assessed by a Kaplan–Meier survival plot, with the hazard ratio (HR) with 95% confidence intervals (CI) and logrank *P* value as previous report [32, 33].

### Statistics

Data between experimental groups were analyzed by the Student′s *t* test or one-way ANOVA. Spearman′s rank test was used to analysis the correlation between miRNAs expression and circHIPK3 levels. *P *< 0.05 was considered statistically significant. All statistical analysis was performed using Statistical Program for Social Sciences (SPSS) software 17.0 (SPSS Inc., Chicago, IL).

## Results

### Identification of differentially expressed miRNAs profiles in GC according to Ming’s classification

**Fig. 1:**
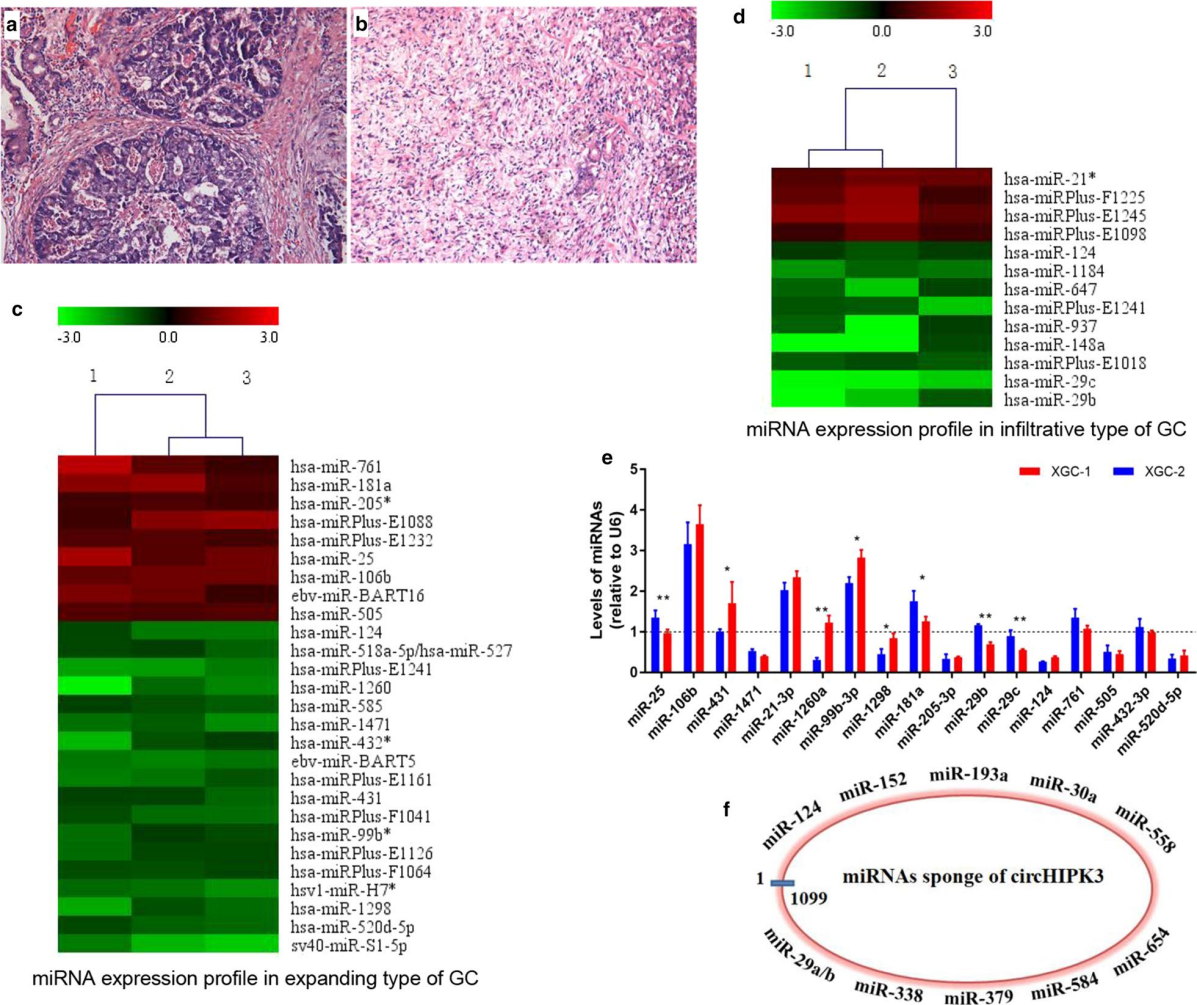
Heat map showing differential miRNA expression profiles between GC tissues and normal gastric tissues according to Ming’s classification. Each column represents a paired sample and each row represents a miRNA. Representative HE staining showed significantly different tumor growth patterns in Ming’s classification of GC (**a** expanding type; **b** infiltrative type). **c**, **d** Hierarchical cluster analysis of the most up and down regulated miRNAs in two types of GC. Each group contains three paired samples. Red strip represents high relative expression and green strip represents low relative expression. **e** Many miRNAs showed differentially expression between Ming’s classification cell lines, GES-1 cell line was used as control. **f** The miRNAs that can be sponged by circHIPK3 according to previous reports

Microarray data showed a significantly differential miRNA expression profiles between two types of GC (Fig. 1, fold change ≥ 1.5, *P* < 0.05). In the infiltrative GC, 13 miRNAs were detected to be differentially regulated, among which 4 miRNAs were up-regulated, while 9 miRNAs were down-regulated compared to their paired normal tissue. 27 miRNAs were differentially expressed in expanding type GC, among which 9 miRNAs were up-regulated and 18 miRNAs down-regulated compared to their paired normal tissue, respectively (Fig. 1, *P* < 0.05). In addition, the expression of some miRNAs associated with Ming’s classification were further verified in Ming’s classification related GC cell lines (XGC-1 and XGC-2), GES-1 was used as control (Fig. 1c). Some miRNAs were consistently down-regulated in microarray data and expression analysis in GC cell line of Ming’s classification. Among them, both of miR-124 and miR-29b can be sponged by circHIPK3 according to previous reports (Fig. 1d) [18, 24, 25].

### MiR-124 and miR-29b were negatively regulated by circHIPK3 in GC cell

**Fig. 2:**
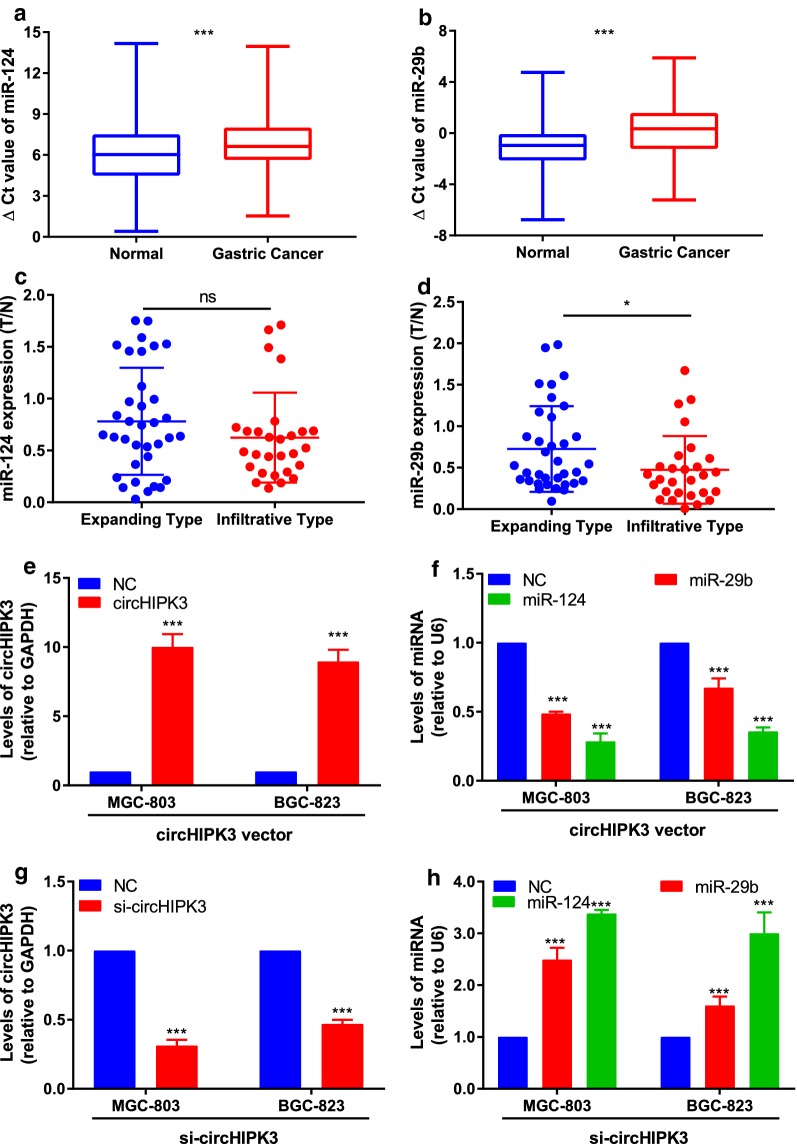
The expression levels of miR-124 and miR-29b in GC samples, and the regulatory role of circHIPK3-miR-124/miR-29b in GC cell. Higher Δ Ct value indicates lower expression. **a**, **b** The expression levels of miR-124 and miR-29b are significantly lower than those in corresponding normal tissues (n = 63, ***P < 0.001). **c**, **d** The association between miR124/miR-29b expression and Ming’s classification in clinical samples. **e**, **g** Expression levels of circHIPK3 was confirmed compared to control in qRT-PCR analysis after transfection. **f**, **h** Expressions of miR-124 and miR-29b were regulated by circHIPK3 compared with negative control. **P < 0.01, ***P < 0.001

With qRT-PCR, we verified that the expression levels of miR-124 and miR-29b were both significantly decreased in human GC tissues compared with their paired normal gastric tissues (Fig. 2a, b, n = 63). The association between their expression and Ming’s classification were also investigated (Fig. 2c, d).

It is reported that circHIPK3 could sponge multiple miRNAs including miR-124/miR-29b in human cancer. Therefore, we suppose that circHIPK3 could play a negative regulatory role on miR-124/miR-29b expression in GC. After transfection with siRNA or circHIPK3 over-expression vector, circHIPK3 expression was identified using qRT-PCR in MGC-803 and BGC-823 cells (Fig. 2e–g). Then, the expressions of miR-124 and miR-29b were found down-regulated significantly in over-expression circHIPK3 group compared with negative control. Accordingly, down-regulation of the circHIPK3 expression could increase the expressions of miR-124 and miR-29b by transfecting GC cells with siRNA (Fig. 2f–h).

### Up-regulation of circHIPK3 associated with clinical aggressive factor and negatively correlated with expression of miR-124 and miR-29b

**Fig. 3:**
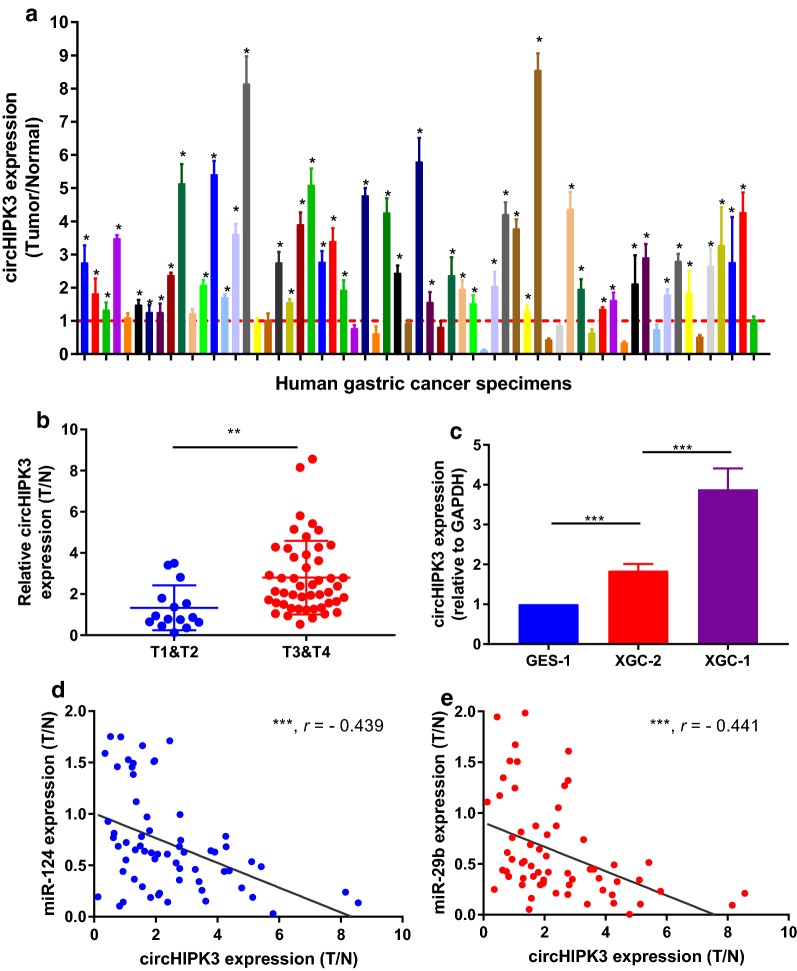
Up-regulation of circHIPK3 correlates with aggressive characteristics of GC. **a** Expression of circHIPK3 in the 63 paired human GC tissues and normal gastric epithelial tissues. **b** Correlation between circHIPK3 expression and T classification in 63 cases with GC. **c** Expression level of circHIPK3 was higher in infiltrative-type GC cell than that in expanding-type GC cell. **d**, **e** Both of miR-124 and miR-29b expression negatively correlated with circHIPK3 expression in GC tissues by qRT-PCR analysis (n = 63, ***P < 0.01)

To explore the expression pattern of circHIPK3 in GC, we detected the expression of circHIPK3 in 63 paired samples diagnosed GC. The result revealed that circHIPK3 level was significantly higher in the GC tissues compared to paired adjacent normal tissues, and closely correlated with T stage and Ming’s classification (Fig. 3a, b and Table 1). Moreover, circHIPK3 expression was higher in infiltrative type GC cell than that in expanding type GC cell (Fig. 3c). These results suggested circHIPK3 expression associated with clinical aggressive factor in GC.

**Table 1:** The correlation between circHIPK3 expression and clinicopathological factors in 63 cases with GC

Characteristics	No. of patients	Mean ± SE	*P* value
Age (years)			
< 60	16	2.59 ± 0.52	0.706
≥ 60	47	2.40 ± 0.24	
Gender			
Male	44	2.54 ± 0.47	0.807
Female	19	2.42 ± 0.25	
Diameter (cm)			
< 5	39	2.21 ± 0.27	0.174
≥ 5	24	2.83 ± 0.37	
Ming’s classification			
Expanding type	35	1.90 ± 0.22	0.005**
Infiltrative type	28	3.14 ± 0.38	
Differentiation			
Low	41	2.56 ± 0.27	0.490
Middle and high	22	2.24 ± 0.40	
Invasion			
T1 and T2	15	1.33 ± 0.28	0.004**
T3 and T4	48	2.80 ± 0.26	
Lymphatic metastasis			
Negative	17	2.23 ± 0.55	0.550
Positive	46	2.53 ± 0.23	
Distal metastasis			
M0	55	2.51 ± 0.24	0.494
M1	8	2.05 ± 0.52	

* *P* < 0.05; ** *P* < 0.01; *** *P* < 0.001

To further examine the relationship between circHIPK3 and miR-124/miR-29b, we analyzed their correlation of expression in the same 63 cases of paired GC tissues. The levels of miR-124 and miR-29b negatively correlated with circHIPK3 expression in GC tissues respectively (Fig. 3d, e). It indicated that circHIPK3 may perform biological roles by miR-124/miR-29b-targets pathways.

### Prediction of circRNA–miRNA pathway

In order to explore the molecular mechanism of circHIPK3-miRNA network, DIANA-miRPath was used to predict the circHIPK3-miR-124/miR-29b-mRNA axis in cancer-related pathways. The result revealed that both miR-124 and miR-29b were associated with cancer-related pathways (Additional file 2: Fig. S1A). The venn diagram demonstrated that there were 18 miR-29b associated pathways, 39 miR-124 associated pathways, and 37 pathways comprised Pathway Union (green) in DIANA-miRPath analysis (Additional file 2: Fig. S1B, C). A total of 8 pathways are the crossing pathways related to miR-29b and miR-124. The statistically significant correlations (*P* value) of these pathways were compared. Among the 8 pathways, the −log2 scaled by P-value of the ECM-receptor interaction showed an infinite value (data not shown in Additional file 2: Fig. S1C). Due to that the study object is GC and the rare reports about pathways of ECM-receptor interaction and Small cell lung cancer in GC, we further focused *pathways in cancer* which also ranked in the top3 for the statistical significance in target prediction (Additional file 2: Fig. S1C).

### Annotation for target genes of circHIPK3-miR-124/miR-29b axes

**Fig. 4:**
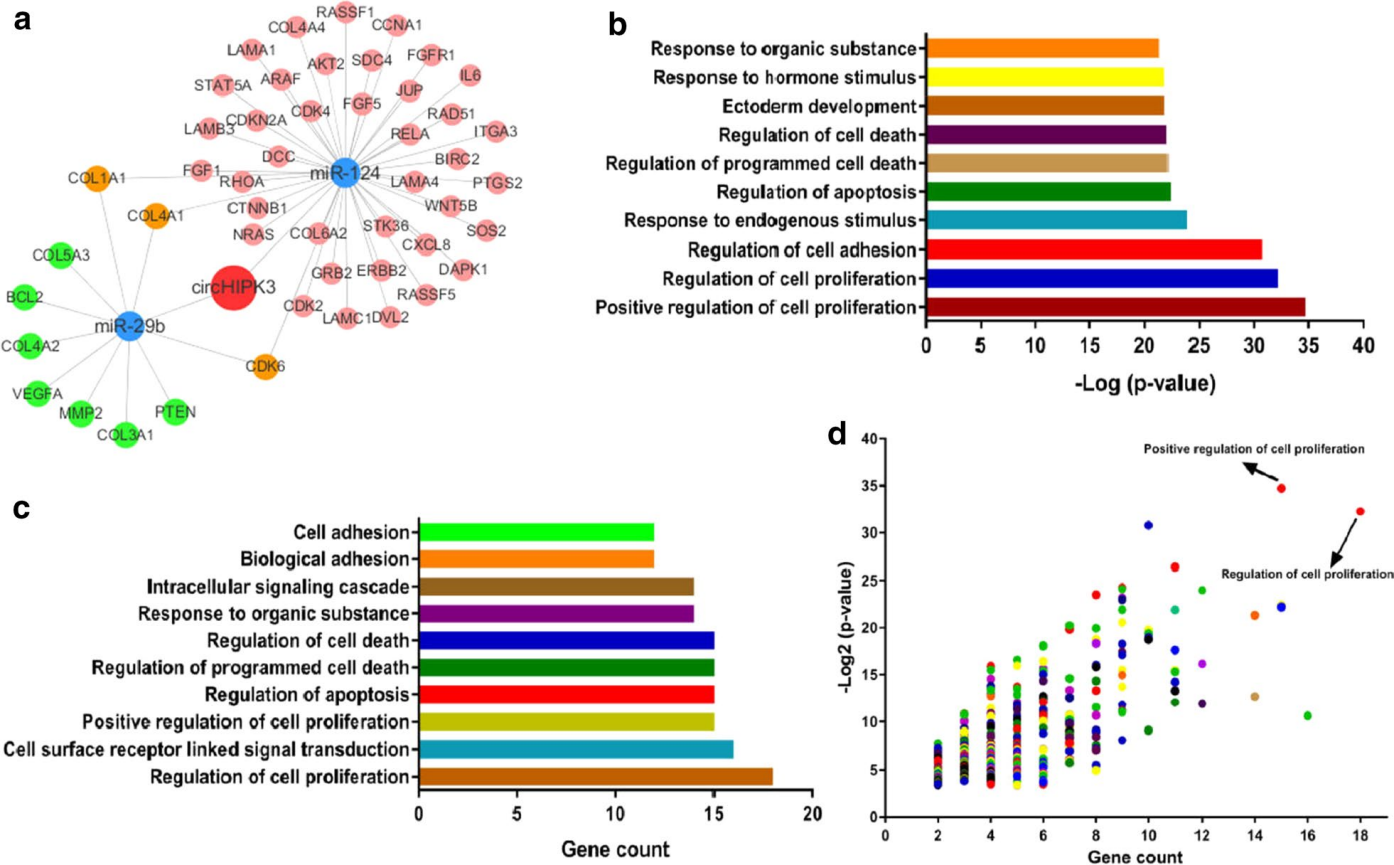
Function annotations for target genes mediated by circHIPK3-miR-124/miR-29b axes in KEGG pathway analysis. **a** Establishment of a circRNA/miRNA/mRNA interactions network of *Pathways in cancer*. **b**, **c** DAVID function annotation for the miR-124 and miR-29b targeted genes of *Pathways in cancer*. The horizontal axes showed *P*-value transformed by −log2 and the gene number of each cluster respectively. The vertical axis shows the annotated functions of the target genes. Only the most significantly enriched clusters were shown. Detail information is in Additional file 4: Table S2. **d** All cluster features about *P*-value and gene count were demonstrated by the scatter plots, and the top right plots represent high significance and more genes. The function of positive regulation of cell proliferation was labeled with high significance and more genes in the combined analysis

DIANA-miRPath was used to predict target genes of miR-124/miR-29b associated with *Pathways in cancer*. The result showed that a total of 51 genes could be regulated by circHIPK3-miR-124/miR-29b axes. Consequently, we established a circRNA/miRNA/mRNA interactions network using Cytoscape (Fig. 4a). Then, the DAVID functional annotation was performed to predict targeted genes as previous report [28, 29]. The analytic result that combined the data of gene count and *P*-value revealed that these genes were significantly associated with cell proliferation (Fig. 4b–d). Then, the data were integrated from KEGG and DIANA-miRPath to draw the cancer-related signaling network including 51 genes of circHIPK3-miR-124/miR-29b axes (Additional file 3: Fig. S2). In the network, Collagen Protein family gene (COL1A1, COL3A1 and COL4A1 et al.) and CDK4/6 could regulate the cell cycle arrest and tumor growth in vivo [34–36]. Most of the targeted genes in the network were considered to be closely related to cell proliferation and tumor growth (Fig. 4 and Additional file 4: Table S2).

### The proliferation of GC cell was positively regulated by circHIPK3

As bioinformatics prediction above, cell growth may be the main function that circHIPK3-miR-124/miR-29b axes regulated. MGC-803 and BGC-823 were widely used materials in GC cell proliferation assay and therefore they were chosen for subsequent cell proliferation assay to investigate the biological role of circHIPK3 using CCK-8 method. GC cells were applied in gain-of-function studies using circHIPK3 over-expression vector, whereas siRNA for circHIPK3 were applied in loss-of-function studies in the experiment. Knockdown of circHIPK3 inhibited cell proliferation, and circHIPK3 over-expression promoted the cell proliferation as shown (Additional file 5: Fig. S3).

### Targets of circHIPK3-miR-124/miR-29b axes up-regulated in GC

**Fig. 5:**
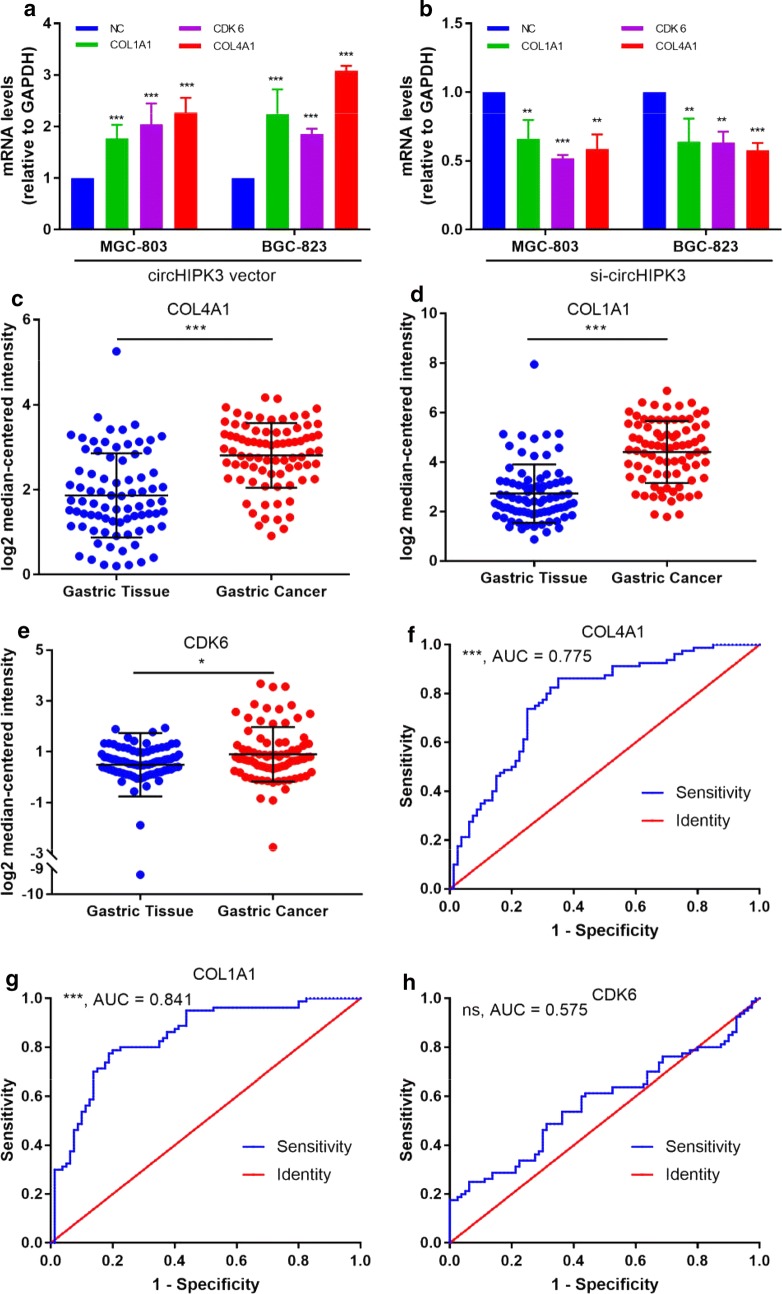
Up-regulated mRNA expression levels of target genes mediated by circHIPK3-miR-124/miR-29b axes revealed by Oncomine analyses in 80 GC cases. **a** Over-expression of circHIPK3 increased the mRNA expression of *COL4A1*, *COL1A1* and *CDK6*. **b** Knockdown of circHIPK3 inhibits mRNA expression of *COL4A1*, *COL1A1* and *CDK6*. **c**–**e** Expressions of COL1A1, COL4A1 and CDK6 were found to be upregulated comparing with normal gastric tissues, respectively. **f**–**h** ROC curve revealed clinical value of COL1A1 and COL4A1 but not CDK6 in the screening of GC. *P < 0.05, **P < 0.01, ***P < 0.001

We then ask if the mRNA levels of these target genes mediated by circHIPK3-miR-124/miR-29b axes were down or up-regulated in GC. As shown (Figs. 4a, 5a, b), three genes (*COL1A1*, *COL4A1* and *CDK6*) could be regulated by both miR-124 and miR-29b in the network, suggesting that they might act crucial roles mediated by circHIPK3-miR-124/miR-29b axes. Then, we used Oncomine cancer microarray mRNA database to indentify the expression levels of these three genes in GC samples. All of these genes showed significantly higher expression in 80 paired cases study (Fig. 5, *P* < 0.05).

Furthermore, the clinical value of COL1A1, COL4A1 and CDK6 were also assessed. ROC curve showed that the status of GC was able to be predicted by using expression levels of COL1A1 and COL4A1 (Fig. 5, area under curve (AUC) = 0.841, *P* < 0.001; AUC = 0.775, *P* < 0.001, respectively) but not CDK6 expression (Fig. 5, AUC = 0.575, *P* = 0.100).

### Targets of circHIPK3-miR-124/miR-29b axes were prognostic markers for survival of GC patients

**Fig. 6:**
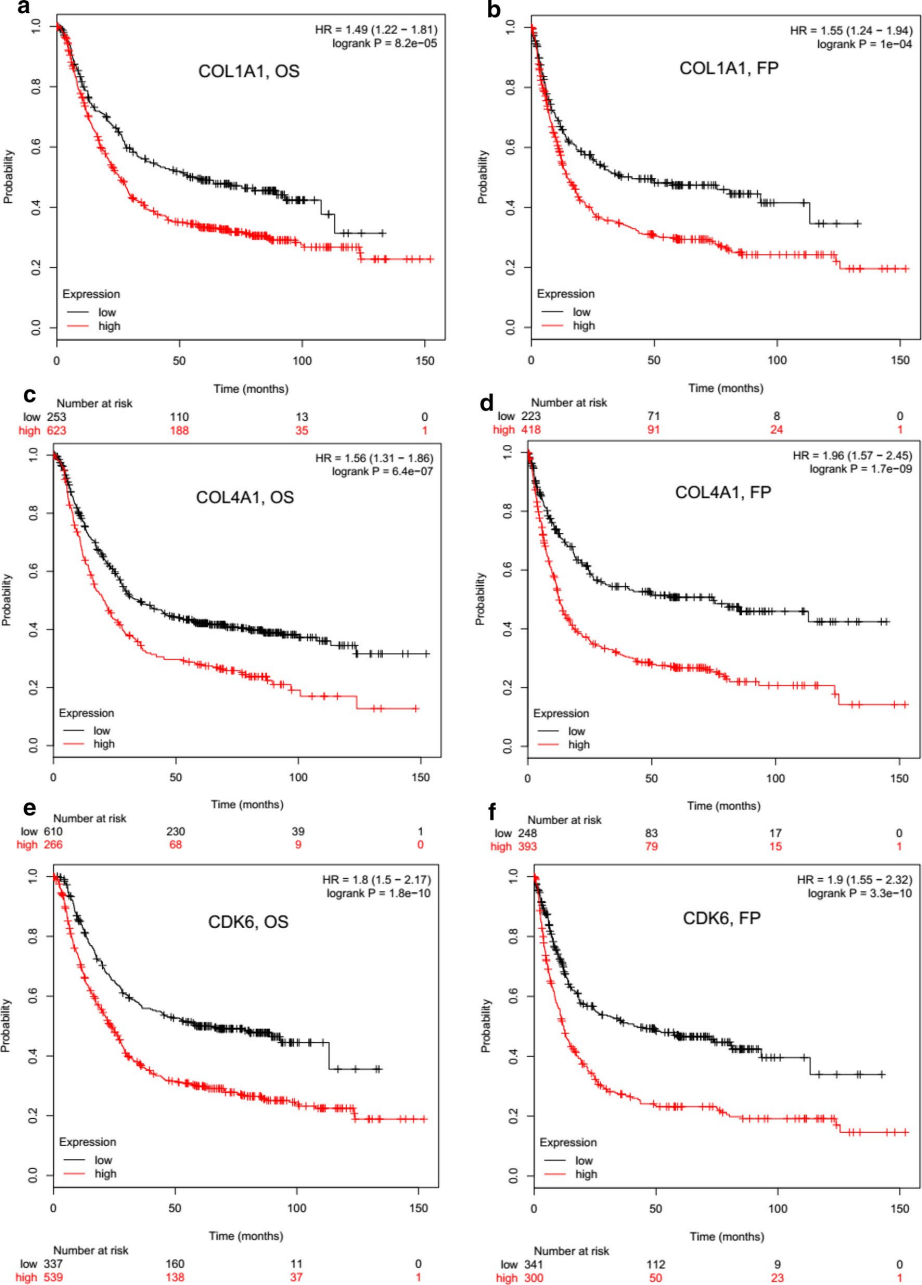
Target genes mediated by circHIPK3-miR-124/miR-29b axes were prognostic markers for survival of patients with GC (n = 876). Higher levels of target gene (**a**, **b**, COL1A1; **c**, **d**, COL4A1 and **e**, **f**, CDK6) was correlated to a poor overall survival (OS) and poor time to first progression (FP) in GC as shown

As the target genes showed aberrantly expression profiles, we asked whether the target genes could serve as prognostic markers in GC patients. Therefore, overall survival (OS) and first progression (FP) curves were plotted by using Kaplan–Meier method according to the gene expression level in 876 GC samples. Patients with higher levels of COL1A1 had a significantly shorter OS (Fig. 6a, logrank *P* = 8.2e−05) and FP (Fig. 6b, logrank *P* = 1e−04) than those with the lower levels of COL1A1. Similar result was also found in survival analysis about mRNA expression of COL4A1 (Fig. 6c, d, OS, logrank *P* = 6.4e−07; FP, logrank *P* = 1.7e−09, respectively) and CDK6 (Fig. 6e, f, OS, logrank *P* = 1.8e−10; FP, logrank *P* = 3.3e−10, respectively).

## Discussion

Although GC has been classified into many subgroups based on the anatomical location, histological types and molecular subtypes, none of these classifications can accurately describe the pathogenesis and the molecular biology of GC [2]. In 1977, Ming provided an original classification based on tumor biological characteristics of growth and invasiveness to evaluate the various aspects of GC [4, 5]. Due to the complex origins of histogenesis in tumor tissue, to date, little has been reported on potential molecular events that can trigger the origin of tumor growth and invasion phenotype in Ming’s classification.

In this study, we first provide the evidence of miRNAs expression profile to understand the molecular mechanism in the Ming’s classification. Within these miRNAs, we found miR-124 and miR-29b were associated with circHIPK3 expression in GC, and knockdown of circHIPK3 suppressed the growth of GC cells. Bioinformatics tools was used to construct circHIPK3-miR124/miR29b network. As a result, three common targets of miR-124 and miR-29b were identified as crucial factors mediated by circHIPK3-miR124/miR29b axes. ROC curve and survival analysis revealed that these three targets were observed as independent prognostic markers in GC patients. These results indicated the specific regulatory network of circRNA–miRNA–mRNA may regulate different histological growing patterns in GC. Furthermore, some targets of this pathway could serve as clinical biomarkers with the value of predicting the GC progression after radical surgery.

It is well known that the deregulation of miRNAs has a crucial role in various human cancers [37]. The aberrant expressions of some miRNAs were found to be associated with tumour classification and clinical outcome [37]. Our previous report clearly demonstrated that miR-145 selectively down-regulated in infiltrative GC compared to expanding GC [4]. Each miRNA may repress up to hundreds of transcripts, which act as letters of a new language in intracellular transcriptive communication [38]. In GC, miR-124 and miR-29b markedly inhibited cancer cell proliferation and tumourigenicity by directly binding the 3′ untranslated regions of their targets respectively [37, 39]. In this study, their expressions are both significantly lower in GC tissues compared that in gastric normal tissues (Fig. 2). The competing endogenous RNA (ceRNA) hypothesis could form a large-scale regulatory network across the transcriptome that greatly expanding the functional genetic information in human carcinogenesis [18, 38]. For example, circMTO1 could suppress human hepatocellular carcinoma progression by acting as the sponge of oncogenic miR-9 to promote p21 expression [40]. Despite lack of confirmation of the interacts between circHIPK3 and miR-124/miR-29b is a limitation in this study, the precise molecular mechanisms of interacts between circHIPK3 and the two microRNAs has been reported in cancer by Zheng et al. [24] and Chen et al. [41], respectively. The circHIPK3 expression is negatively associated with the expression miR-124/miR-29b in GC samples (Fig. 3). In addition, our results suggested that circHIPK3 was up-regulated along with the progression of T stage in clinical samples and showed higher expression in infiltrative-type GC cell than that in expanding-type GC cell (Fig. 3 and Table 1). Collectively, the discovery that circRNA function as efficient miRNAs sponges provide new insight to explore the pathophysiologic mechanism in cancer development.

**Fig. 7:**
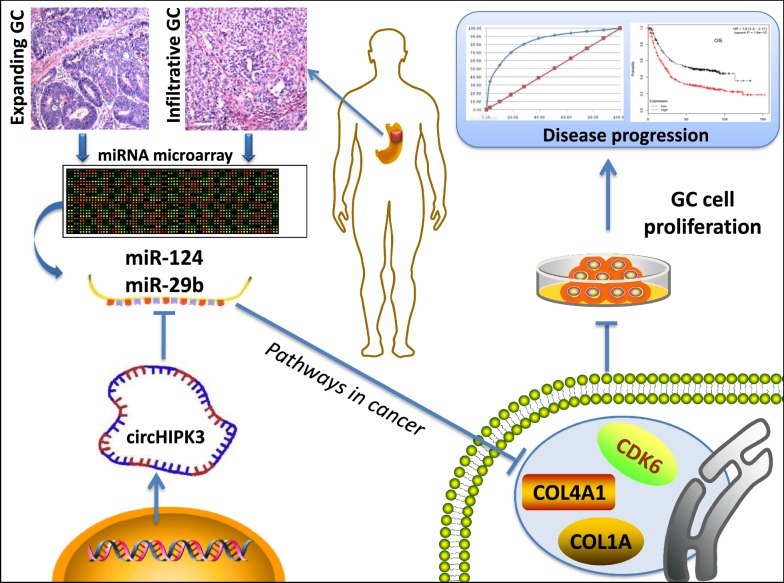
Proposed scheme for the roles of circHIPK3-miR-124/miR-29b axes on tumor growth and clinical progression in GC. Significantly differential expressed miRNAs were found in two pathological types of GC. Both of miR-124 and miR-29b were consistently down-regulated in GC. CircHIPK3 acted a negative regulatory role on miR-124/miR-29b expression and inhibited GC cell proliferation. The bioinformatics analyses showed that targets expression of circHIPK3-miR-124/miR-29b axes in *Pathways in cancer* was able to predict the status of GC and associated with individual survival time

Due to a large number of targets of miRNA, we performed bioinformatics approaches to screening the common targets in the circHIPK3-miR124/miR29b axes. COL1A1 and COL4A1 are family members of human collagen genes. COL1A1 encodes the major component of fibrillar collagen found in most connective tissues, and involved in gap junction, cell proliferation and tumor invasion [42, 43]. COL4A1 is an important flexible protein in the structure of the basement membranes interacted with nearby cells, playing a role in cell migration and growth [44]. It suggested that altered expression levels of these collagens may contribute to the formation of infiltrative growth patterns in human cancer development [44]. We reported expression levels of COL1A1 and COL4A1 were prognostic biomarkers for survival of patients with GC (Fig. 6). Cyclin-dependent kinases 6 (CDK6) is a protein kinase activating cell proliferation, and involved in the restriction of the cell cycle [45]. Recently, up-regulated circRNA_100290 was discovered co-expressed with CDK6 by sponging miR-29b in oral squamous cell carcinomas tissue [46]. It is indicated that CDK6 maybe a direct target in the circRNA–miRNA regulatory network of human cancer, and also served as a prognostic biomarker of GC in this study. All the above evidences imply that functional annotation could been used as a powerful tool to screen and identify the potential factors involved in GC development. A model was proposed for the roles of circHIPK3-miR-124/miR-29b axes in GC progression (Fig. 7).

## Conclusions

In summary, our work demonstrates that the targets of circHIPK3-miR-124/miR-29b axes has relevance to progression of GC. The systematic identification and characterization of circHIPK3 regulatory network in gene expression pattern is helpful to expand our understanding of the transcription mechanisms in the histopathological origin of GC. This study may provide new insights and therapeutic strategies for cancer prevention and treatment in future.

## Additional files

**Additional file 1: Table S1.** The sequence of primers and siRNA.

**Additional file 2: Fig. S1.** Prediction of circHIPK3- miR-124/miR-29b pathway. (A) Statistically significant correlations were revealed between miR-29b or miR-124 and their mediated pathways by *P*-value (log scaled) in heatmap. Red represents high significance. (B) The relevant pathways of miR-29b mediated, miR-124 mediated, and miR-29b and miR-124 jointly mediated. (C) The magnitudes of the significant correlation about Pathways among miR-29b mediated, miR-124 mediated, and miR-29b and miR-124 jointly mediated by P-value (−log2 scaled).

**Additional file 3: Fig. S2.** Mapping of *Pathways in cancer* mediated by miR-124 and miR-29b. Yellow marked nodes are associated with target genes regulated by circHIPK3-miR-124/miR-29b axes enrichment on this pathway and devoted to the initiation and progression of GC.

**Additional file 4: Table S2.** Detail information of enriched clusters.

**Additional file 5: Fig. S3.** Effect of circHIPK3 on cell proliferation. (A, B) Knockdown of circHIPK3 inhibits human GC cell proliferation. (C, D) Over-expression of circHIPK3 promoted the cell proliferation. NC, negative control. *P < 0.05, **P < 0.01, ***P < 0.001.

## Abbreviations

GC: gastric cancer
HIPK3: homeodomain-interacting protein kinase-3
circHIPK3: circRNA 0000284
miRNAs: microRNAs
circRNA: circular RNA
ceRNA: competing endogenous RNA
f-circRNA: fusion circRNAs
TNM: tumor-node metastasis
NCCN: National Comprehensive Cancer Network
qRT-PCR: quantitative reverse transcription polymerase chain reaction
OD: optical density
ECM: extracellular matrix
CDK6: cyclin-dependent kinases 6
CCK-8: Cell Counting Kit-8
COL1A1: collagen type I alpha 1 chain
COL4A1: collagen type IV alpha 1 chain
HR: hazard ratio
ROC: receiver operating characteristic
OS: overall survival
FP: first progression
CI: confidence intervals
SPSS: Statistical Program for Social Sciences
