# Supplementary material for: Regulatory network of circRNA–miRNA–mRNA contributes to the histological classification and disease progression in gastric cancer
Source: J Transl Med. 2018 Aug 2;16:216. doi: 10.1186/s12967-018-1582-8 (PMC6071397; doi:10.1186/s12967-018-1582-8)
Supplement: Supplementary file 1 — Additional file 1: Table S1. The sequence of primers and siRNA. [file 12967_2018_1582_MOESM1_ESM.docx]

| Supplementary Table 1. The sequence of primers and siRNA | |
| --- | --- |
| CircHIPK3-F | 5-TTGGTGGATCCTGTTCGGC-3 |
| CircHIPK3-R | 5- GTAGACCAAGACTTGTGAGGC-3 |
| GAPDH-F | 5-AGAAGGCTGGGGCTCATTTG-3 |
| GAPDH-R | 5-AGGGGCCATCCACAGTCTTC-3 |
| COL1A1-F | 5-GAGGGCCAAGACGAAGACATC-3 |
| COL1A1-R | 5-CAGATCACGTCATCGCACAAC-3 |
| CDK6 primer-F | 5-TGCACAGTGTCACGAACAGA-3 |
| CDK6 primer-R | 5-ACCTCGGAGAAGCTGAAACA-3 |
| COL4A1-F | 5-CCAGGGGTCGGAGAGAAAG-3 |
| COL4A1-R | 5-GGTCCTGTGCCTATAACAATTCC-3 |
| si-circHIPK3 | 5-CUACAGGUAUGGCCUCACA-3 |
