# Supplementary figures and images for: Regulatory network of circRNA–miRNA–mRNA contributes to the histological classification and disease progression in gastric cancer
Source: J Transl Med. 2018 Aug 2;16:216. doi: 10.1186/s12967-018-1582-8 (PMC6071397; doi:10.1186/s12967-018-1582-8)

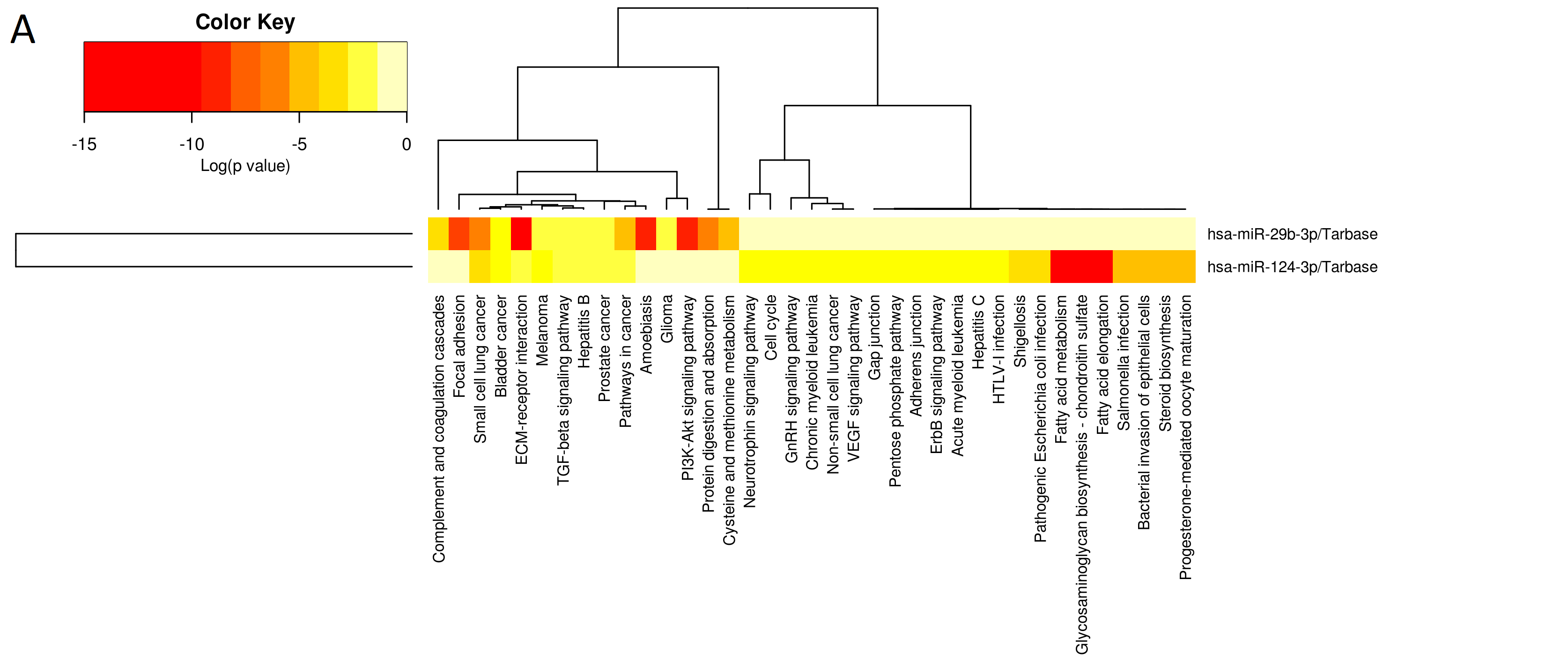

Supplement: Supplementary file 2 — Additional file 2: Fig. S1. Prediction of circHIPK3- miR-124/miR-29b pathway. (A) Statistically significant correlations were revealed between miR-29b or miR-124 and their mediated pathways by P-value (log scaled) in heatmap. Red represents high significance. (B) The relevant pathways of miR-29b mediated, miR-124 mediated, and miR-29b and miR-124 jointly mediated. (C) The magnitudes of the significant correlation about Pathways among miR-29b mediated, miR-124 mediated, and miR-29b and miR-124 jointly mediated by P-value (−log2 scaled). [file 12967_2018_1582_MOESM2_ESM.zip › Figure S1A.tif]

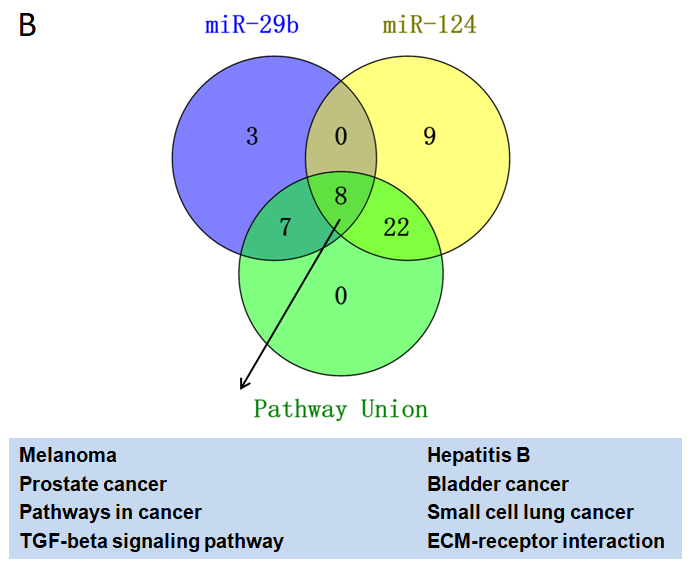

Supplement: Supplementary file 2 — Additional file 2: Fig. S1. Prediction of circHIPK3- miR-124/miR-29b pathway. (A) Statistically significant correlations were revealed between miR-29b or miR-124 and their mediated pathways by P-value (log scaled) in heatmap. Red represents high significance. (B) The relevant pathways of miR-29b mediated, miR-124 mediated, and miR-29b and miR-124 jointly mediated. (C) The magnitudes of the significant correlation about Pathways among miR-29b mediated, miR-124 mediated, and miR-29b and miR-124 jointly mediated by P-value (−log2 scaled). [file 12967_2018_1582_MOESM2_ESM.zip › Figure S1B.tif]

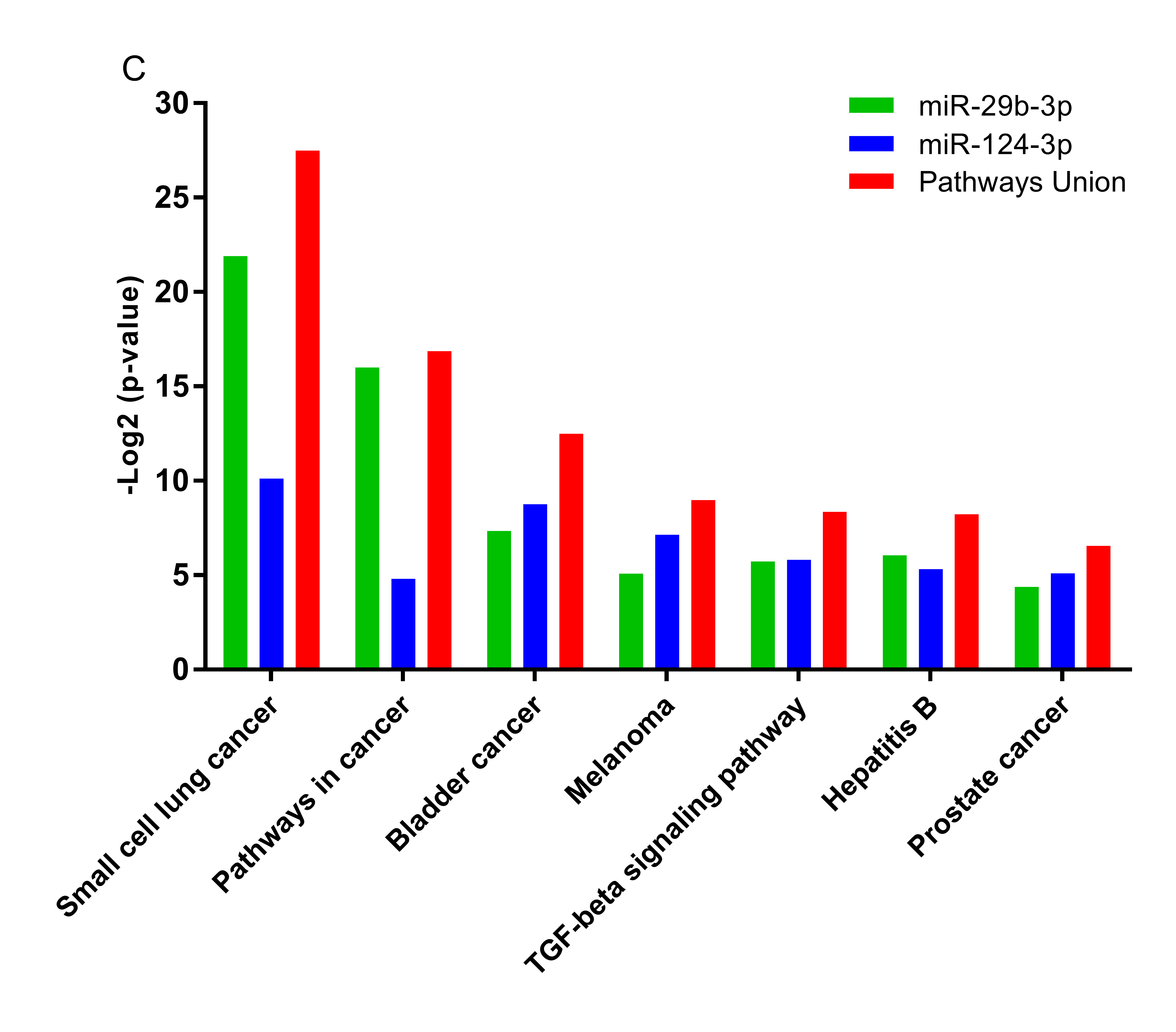

Supplement: Supplementary file 2 — Additional file 2: Fig. S1. Prediction of circHIPK3- miR-124/miR-29b pathway. (A) Statistically significant correlations were revealed between miR-29b or miR-124 and their mediated pathways by P-value (log scaled) in heatmap. Red represents high significance. (B) The relevant pathways of miR-29b mediated, miR-124 mediated, and miR-29b and miR-124 jointly mediated. (C) The magnitudes of the significant correlation about Pathways among miR-29b mediated, miR-124 mediated, and miR-29b and miR-124 jointly mediated by P-value (−log2 scaled). [file 12967_2018_1582_MOESM2_ESM.zip › Figure S1C.tif]

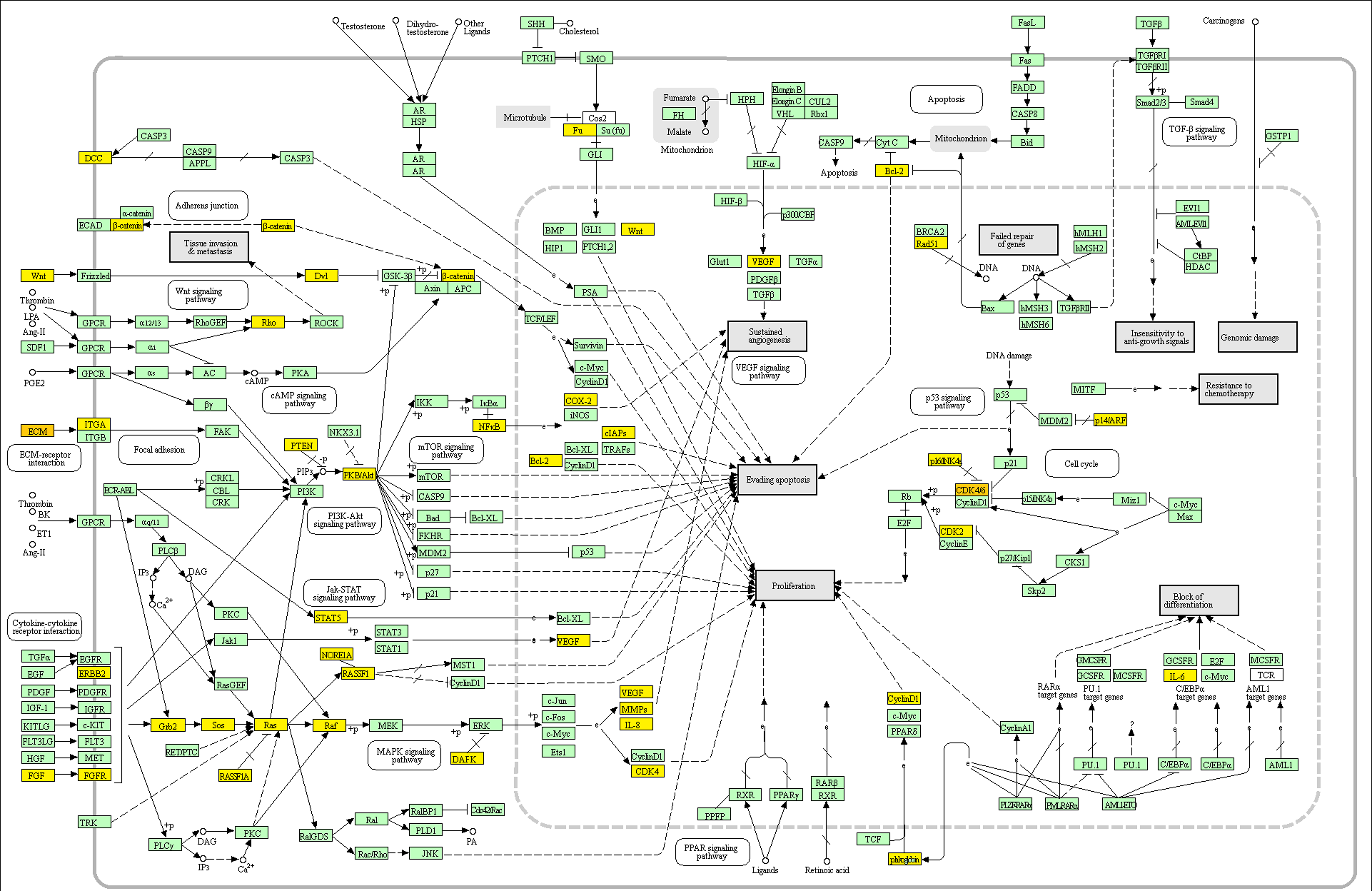

Supplement: Supplementary file 3 — Additional file 3: Fig. S2. Mapping of Pathways in cancer mediated by miR-124 and miR-29b. Yellow marked nodes are associated with target genes regulated by circHIPK3-miR-124/miR-29b axes enrichment on this pathway and devoted to the initiation and progression of GC. [file 12967_2018_1582_MOESM3_ESM.tif]

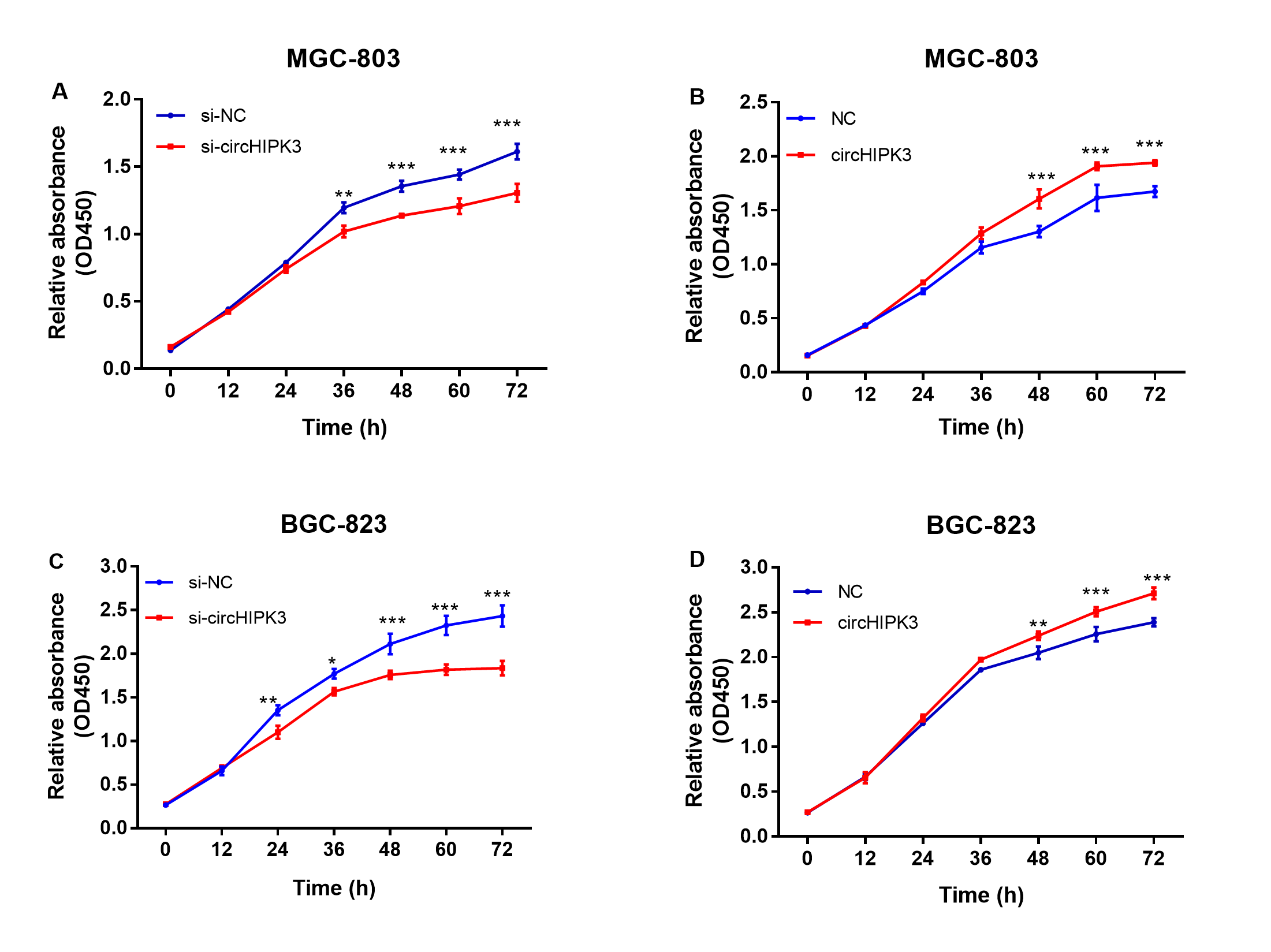

Supplement: Supplementary file 5 — Additional file 5: Fig. S3. Effect of circHIPK3 on cell proliferation. (A, B) Knockdown of circHIPK3 inhibits human GC cell proliferation. (C, D) Over-expression of circHIPK3 promoted the cell proliferation. NC, negative control. *P < 0.05, **P < 0.01, ***P < 0.001. [file 12967_2018_1582_MOESM5_ESM.tif]
