# Supplementary material for: Regulatory network of circRNA–miRNA–mRNA contributes to the histological classification and disease progression in gastric cancer
Source: J Transl Med. 2018 Aug 2;16:216. doi: 10.1186/s12967-018-1582-8 (PMC6071397; doi:10.1186/s12967-018-1582-8)
Supplement: Supplementary file 4 — Additional file 4: Table S2. Detail information of enriched clusters. [file 12967_2018_1582_MOESM4_ESM.docx]

| **Supplementary Table 2** | **Detail information of enriched clusters** |  |  |  |  |  |  |  |  |  |  |  |  |
| --- | --- | --- | --- | --- | --- | --- | --- | --- | --- | --- | --- | --- | --- |
| Category | Term | Count | % | PValue | log | Genes | List Total | Pop Hits | Pop Total | Fold Enrichment | Bonferroni | Benjamini | FDR |
| GOTERM_BP_FAT | GO:0042127~regulation of cell proliferation | 18.00 | 2.43 | 0.00 | 32.22 | 779920, 803206, 799969, 783742, 781709, 819273, 823371, 817755, 815476, 818089, 797559, 781362, 825577, 796239, 799249, 810015, 798079, 781132 | 46 | 787 | 13528 | 6.726258 | 2.64E-07 | 1.32E-07 | 3.27E-07 |
| GOTERM_BP_FAT | GO:0007166~cell surface receptor linked signal transduction | 16.00 | 2.16 | 0.00 | 10.59 | 779920, 783742, 781709, 819273, 795569, 817755, 784852, 815476, 779314, 816636, 799249, 775320, 787106, 796732, 804749, 781132 | 46 | 1856 | 13528 | 2.535232 | 0.576254 | 0.014951 | 1.057725 |
| GOTERM_BP_FAT | GO:0008284~positive regulation of cell proliferation | 15.00 | 2.02 | 0.00 | 34.71 | 803206, 799969, 783742, 781709, 819273, 823371, 817755, 815476, 818089, 797559, 825577, 796239, 799249, 810015, 798079 | 46 | 414 | 13528 | 10.65532 | 4.72E-08 | 4.72E-08 | 5.84E-08 |
| GOTERM_BP_FAT | GO:0042981~regulation of apoptosis | 15.00 | 2.02 | 0.00 | 22.39 | 779920, 799969, 814431, 783742, 781709, 819273, 785012, 784852, 818089, 781362, 786559, 825577, 796239, 810015, 819639 | 46 | 804 | 13528 | 5.486697 | 2.40E-04 | 4.80E-05 | 2.97E-04 |
| GOTERM_BP_FAT | GO:0043067~regulation of programmed cell death | 15.00 | 2.02 | 0.00 | 22.22 | 779920, 799969, 814431, 783742, 781709, 819273, 785012, 784852, 818089, 781362, 786559, 825577, 796239, 810015, 819639 | 46 | 812 | 13528 | 5.432641 | 2.71E-04 | 4.52E-05 | 3.36E-04 |
| GOTERM_BP_FAT | GO:0010941~regulation of cell death | 15.00 | 2.02 | 0.00 | 22.15 | 779920, 799969, 814431, 783742, 781709, 819273, 785012, 784852, 818089, 781362, 786559, 825577, 796239, 810015, 819639 | 46 | 815 | 13528 | 5.412643 | 2.84E-04 | 4.05E-05 | 3.51E-04 |
| GOTERM_BP_FAT | GO:0010033~response to organic substance | 14.00 | 1.89 | 0.00 | 21.34 | 779920, 799969, 783742, 819273, 795569, 781978, 784852, 779314, 818089, 825577, 810015, 796732, 781132, 808600 | 46 | 721 | 13528 | 5.710426 | 4.96E-04 | 4.96E-05 | 6.15E-04 |
| GOTERM_BP_FAT | GO:0007242~intracellular signaling cascade | 14.00 | 1.89 | 0.00 | 12.66 | 814431, 783742, 819273, 817755, 815476, 779314, 794222, 814239, 786559, 796239, 775320, 796706, 819639, 781132 | 46 | 1256 | 13528 | 3.278039 | 0.184434 | 0.00473 | 0.252168 |
| GOTERM_BP_FAT | GO:0009719~response to endogenous stimulus | 12.00 | 1.62 | 0.00 | 23.96 | 779920, 799969, 783742, 825577, 819273, 810015, 796732, 784852, 779314, 781132, 818089, 808600 | 46 | 405 | 13528 | 8.713688 | 8.11E-05 | 2.03E-05 | 1.00E-04 |
| GOTERM_BP_FAT | GO:0007155~cell adhesion | 12.00 | 1.62 | 0.00 | 16.20 | 799969, 814431, 823371, 826660, 795569, 799249, 787106, 781978, 822774, 822268, 781132, 810626 | 46 | 700 | 13528 | 5.041491 | 0.017401 | 7.31E-04 | 0.021738 |
| GOTERM_BP_FAT | GO:0022610~biological adhesion | 12.00 | 1.62 | 0.00 | 16.18 | 799969, 814431, 823371, 826660, 795569, 799249, 787106, 781978, 822774, 822268, 781132, 810626 | 46 | 701 | 13528 | 5.034299 | 0.017634 | 7.11E-04 | 0.022032 |
| GOTERM_BP_FAT | GO:0006796~phosphate metabolic process | 12.00 | 1.62 | 0.00 | 11.89 | 803206, 779920, 799969, 783742, 819273, 796706, 796732, 798079, 815476, 819639, 806167, 797559 | 46 | 973 | 13528 | 3.626972 | 0.294209 | 0.007386 | 0.43059 |
| GOTERM_BP_FAT | GO:0006793~phosphorus metabolic process | 12.00 | 1.62 | 0.00 | 11.89 | 803206, 779920, 799969, 783742, 819273, 796706, 796732, 798079, 815476, 819639, 806167, 797559 | 46 | 973 | 13528 | 3.626972 | 0.294209 | 0.007386 | 0.43059 |
| GOTERM_BP_FAT | GO:0009725~response to hormone stimulus | 11.00 | 1.48 | 0.00 | 21.87 | 779920, 799969, 783742, 825577, 819273, 810015, 796732, 779314, 781132, 818089, 808600 | 46 | 367 | 13528 | 8.814595 | 3.45E-04 | 3.84E-05 | 4.28E-04 |
| GOTERM_BP_FAT | GO:0006915~apoptosis | 11.00 | 1.48 | 0.00 | 15.47 | 779920, 781362, 799969, 786559, 825577, 796239, 785012, 784852, 819639, 781132, 794222 | 46 | 602 | 13528 | 5.373682 | 0.028614 | 9.67E-04 | 0.035947 |
| GOTERM_BP_FAT | GO:0012501~programmed cell death | 11.00 | 1.48 | 0.00 | 15.29 | 779920, 781362, 799969, 786559, 825577, 796239, 785012, 784852, 819639, 781132, 794222 | 46 | 611 | 13528 | 5.294528 | 0.032448 | 0.00103 | 0.040843 |
| GOTERM_BP_FAT | GO:0006468~protein amino acid phosphorylation | 11.00 | 1.48 | 0.00 | 14.21 | 803206, 799969, 783742, 819273, 796706, 796732, 798079, 815476, 819639, 806167, 797559 | 46 | 667 | 13528 | 4.85001 | 0.06728 | 0.001933 | 0.086221 |
| GOTERM_BP_FAT | GO:0008219~cell death | 11.00 | 1.48 | 0.00 | 13.30 | 779920, 781362, 799969, 786559, 825577, 796239, 785012, 784852, 819639, 781132, 794222 | 46 | 719 | 13528 | 4.499244 | 0.122503 | 0.003433 | 0.161712 |
| GOTERM_BP_FAT | GO:0016265~death | 11.00 | 1.48 | 0.00 | 13.22 | 779920, 781362, 799969, 786559, 825577, 796239, 785012, 784852, 819639, 781132, 794222 | 46 | 724 | 13528 | 4.468172 | 0.129273 | 0.003455 | 0.171287 |
| GOTERM_BP_FAT | GO:0016310~phosphorylation | 11.00 | 1.48 | 0.00 | 12.04 | 803206, 799969, 783742, 819273, 796706, 796732, 798079, 815476, 819639, 806167, 797559 | 46 | 800 | 13528 | 4.043696 | 0.269466 | 0.006802 | 0.388093 |
| GOTERM_BP_FAT | GO:0030155~regulation of cell adhesion | 10.00 | 1.35 | 0.00 | 30.74 | 779920, 781362, 799969, 783742, 826660, 819273, 799249, 817755, 798079, 808600 | 46 | 137 | 13528 | 21.4662 | 7.39E-07 | 2.46E-07 | 9.15E-07 |
| GOTERM_BP_FAT | GO:0010647~positive regulation of cell communication | 10.00 | 1.35 | 0.00 | 19.80 | 819591, 814431, 825577, 781709, 796239, 819273, 810015, 784852, 781132, 818089 | 46 | 329 | 13528 | 8.938813 | 0.001443 | 1.03E-04 | 0.001788 |
| GOTERM_BP_FAT | GO:0007167~enzyme linked receptor protein signaling pathway | 10.00 | 1.35 | 0.00 | 19.34 | 779920, 783742, 816636, 781709, 819273, 795569, 796732, 817755, 815476, 779314 | 46 | 342 | 13528 | 8.599034 | 0.001989 | 1.33E-04 | 0.002466 |
| GOTERM_BP_FAT | GO:0043066~negative regulation of apoptosis | 10.00 | 1.35 | 0.00 | 18.93 | 779920, 799969, 814431, 783742, 825577, 781709, 796239, 819273, 819639, 818089 | 46 | 354 | 13528 | 8.307541 | 0.002644 | 1.65E-04 | 0.003279 |
| GOTERM_BP_FAT | GO:0043069~negative regulation of programmed cell death | 10.00 | 1.35 | 0.00 | 18.76 | 779920, 799969, 814431, 783742, 825577, 781709, 796239, 819273, 819639, 818089 | 46 | 359 | 13528 | 8.191837 | 0.002967 | 1.75E-04 | 0.00368 |
| GOTERM_BP_FAT | GO:0060548~negative regulation of cell death | 10.00 | 1.35 | 0.00 | 18.73 | 779920, 799969, 814431, 783742, 825577, 781709, 796239, 819273, 819639, 818089 | 46 | 360 | 13528 | 8.169082 | 0.003036 | 1.60E-04 | 0.003766 |
| GOTERM_BP_FAT | GO:0010604~positive regulation of macromolecule metabolic process | 10.00 | 1.35 | 0.00 | 9.14 | 803206, 799969, 783742, 825577, 781709, 819016, 796732, 781132, 818089, 797559 | 46 | 857 | 13528 | 3.431586 | 0.903439 | 0.032843 | 2.853469 |
| GOTERM_BP_FAT | GO:0007398~ectoderm development | 9.00 | 1.21 | 0.00 | 21.89 | 781362, 799969, 795569, 810015, 822774, 781132, 818089, 810626, 808600 | 46 | 199 | 13528 | 13.30042 | 3.40E-04 | 4.25E-05 | 4.21E-04 |
| GOTERM_BP_FAT | GO:0007169~transmembrane receptor protein tyrosine kinase signaling pathway | 9.00 | 1.21 | 0.00 | 20.59 | 779920, 783742, 816636, 781709, 819273, 796732, 817755, 815476, 779314 | 46 | 224 | 13528 | 11.81599 | 8.38E-04 | 7.62E-05 | 0.001038 |
| GOTERM_BP_FAT | GO:0022402~cell cycle process | 9.00 | 1.21 | 0.00 | 11.01 | 803206, 781362, 799969, 814239, 819016, 798079, 781132, 798174, 797559 | 46 | 565 | 13528 | 4.684571 | 0.472389 | 0.012221 | 0.788732 |
| GOTERM_BP_FAT | GO:0007049~cell cycle | 9.00 | 1.21 | 0.00 | 8.09 | 803206, 781362, 799969, 814239, 819016, 798079, 781132, 798174, 797559 | 46 | 776 | 13528 | 3.410802 | 0.992301 | 0.055017 | 5.849005 |
| GOTERM_BP_FAT | GO:0043062~extracellular structure organization | 8.00 | 1.08 | 0.00 | 19.93 | 823371, 819273, 795569, 781978, 781132, 810626, 808600, 804645 | 46 | 163 | 13528 | 14.43372 | 0.001321 | 1.02E-04 | 0.001637 |
| GOTERM_BP_FAT | GO:0008544~epidermis development | 8.00 | 1.08 | 0.00 | 18.76 | 781362, 799969, 795569, 810015, 822774, 818089, 810626, 808600 | 46 | 184 | 13528 | 12.78639 | 0.002972 | 1.65E-04 | 0.003686 |
| GOTERM_BP_FAT | GO:0048545~response to steroid hormone stimulus | 8.00 | 1.08 | 0.00 | 18.35 | 779920, 799969, 825577, 819273, 810015, 781132, 818089, 808600 | 46 | 192 | 13528 | 12.25362 | 0.003942 | 1.97E-04 | 0.004892 |
| GOTERM_BP_FAT | GO:0001568~blood vessel development | 8.00 | 1.08 | 0.00 | 16.04 | 779920, 781709, 826660, 795569, 798126, 817755, 781132, 808600 | 46 | 245 | 13528 | 9.602839 | 0.019361 | 7.52E-04 | 0.02421 |
| GOTERM_BP_FAT | GO:0010627~regulation of protein kinase cascade | 8.00 | 1.08 | 0.00 | 15.89 | 779920, 814431, 825577, 819273, 796732, 784852, 781132, 818089 | 46 | 249 | 13528 | 9.448577 | 0.021478 | 7.75E-04 | 0.026886 |
| GOTERM_BP_FAT | GO:0001944~vasculature development | 8.00 | 1.08 | 0.00 | 15.82 | 779920, 781709, 826660, 795569, 798126, 817755, 781132, 808600 | 46 | 251 | 13528 | 9.373289 | 0.022606 | 7.88E-04 | 0.028313 |
| GOTERM_BP_FAT | GO:0009967~positive regulation of signal transduction | 8.00 | 1.08 | 0.00 | 14.33 | 814431, 825577, 781709, 796239, 819273, 784852, 781132, 818089 | 46 | 295 | 13528 | 7.975239 | 0.0623 | 0.001836 | 0.079632 |
| GOTERM_BP_FAT | GO:0051726~regulation of cell cycle | 8.00 | 1.08 | 0.00 | 13.28 | 803206, 779920, 781362, 799969, 783742, 810015, 798079, 797559 | 46 | 331 | 13528 | 7.107842 | 0.124329 | 0.003398 | 0.164287 |
| GOTERM_BP_FAT | GO:0010557~positive regulation of macromolecule biosynthetic process | 8.00 | 1.08 | 0.01 | 7.52 | 803206, 783742, 825577, 781709, 796732, 781132, 818089, 797559 | 46 | 654 | 13528 | 3.597394 | 0.99928 | 0.071173 | 8.571227 |
| GOTERM_BP_FAT | GO:0031328~positive regulation of cellular biosynthetic process | 8.00 | 1.08 | 0.01 | 7.16 | 803206, 783742, 825577, 781709, 796732, 781132, 818089, 797559 | 46 | 685 | 13528 | 3.434592 | 0.999907 | 0.080931 | 10.86062 |
| GOTERM_BP_FAT | GO:0009891~positive regulation of biosynthetic process | 8.00 | 1.08 | 0.01 | 7.05 | 803206, 783742, 825577, 781709, 796732, 781132, 818089, 797559 | 46 | 695 | 13528 | 3.385174 | 0.999956 | 0.083514 | 11.68004 |
| GOTERM_BP_FAT | GO:0031589~cell-substrate adhesion | 7.00 | 0.94 | 0.00 | 20.25 | 799969, 814431, 823371, 795569, 787106, 781132, 810626 | 46 | 98 | 13528 | 21.00621 | 0.00106 | 8.84E-05 | 0.001313 |
| GOTERM_BP_FAT | GO:0001501~skeletal system development | 7.00 | 0.94 | 0.00 | 10.67 | 799969, 795569, 798126, 810015, 815476, 781132, 808600 | 46 | 319 | 13528 | 6.453319 | 0.556611 | 0.014679 | 1.002183 |
| GOTERM_BP_FAT | GO:0048666~neuron development | 7.00 | 0.94 | 0.00 | 10.21 | 779920, 799969, 825577, 781709, 819273, 785012, 815476 | 46 | 339 | 13528 | 6.072592 | 0.673055 | 0.01846 | 1.375 |
| GOTERM_BP_FAT | GO:0000902~cell morphogenesis | 7.00 | 0.94 | 0.00 | 9.84 | 799969, 814431, 823371, 819273, 785012, 799249, 781132 | 46 | 356 | 13528 | 5.782609 | 0.763253 | 0.02297 | 1.768487 |
| GOTERM_BP_FAT | GO:0008285~negative regulation of cell proliferation | 7.00 | 0.94 | 0.00 | 9.74 | 779920, 781362, 799969, 825577, 819273, 810015, 798079 | 46 | 361 | 13528 | 5.702517 | 0.787366 | 0.0239 | 1.899081 |
| GOTERM_BP_FAT | GO:0032989~cellular component morphogenesis | 7.00 | 0.94 | 0.00 | 9.04 | 799969, 814431, 823371, 819273, 785012, 799249, 781132 | 46 | 397 | 13528 | 5.185412 | 0.91914 | 0.034803 | 3.066739 |
| GOTERM_BP_FAT | GO:0022403~cell cycle phase | 7.00 | 0.94 | 0.00 | 8.73 | 803206, 781362, 799969, 819016, 798079, 798174, 797559 | 46 | 414 | 13528 | 4.972485 | 0.955241 | 0.040573 | 3.774121 |
| GOTERM_BP_FAT | GO:0043065~positive regulation of apoptosis | 7.00 | 0.94 | 0.00 | 8.46 | 779920, 781362, 799969, 786559, 785012, 810015, 819639 | 46 | 430 | 13528 | 4.787462 | 0.976584 | 0.045845 | 4.543119 |
| GOTERM_BP_FAT | GO:0043068~positive regulation of programmed cell death | 7.00 | 0.94 | 0.00 | 8.41 | 779920, 781362, 799969, 786559, 785012, 810015, 819639 | 46 | 433 | 13528 | 4.754293 | 0.979481 | 0.046847 | 4.699093 |
| GOTERM_BP_FAT | GO:0010942~positive regulation of cell death | 7.00 | 0.94 | 0.00 | 8.38 | 779920, 781362, 799969, 786559, 785012, 810015, 819639 | 46 | 435 | 13528 | 4.732434 | 0.981246 | 0.046778 | 4.805208 |
| GOTERM_BP_FAT | GO:0030182~neuron differentiation | 7.00 | 0.94 | 0.00 | 8.33 | 779920, 799969, 825577, 781709, 819273, 785012, 815476 | 46 | 438 | 13528 | 4.70002 | 0.983661 | 0.047799 | 4.967614 |
| GOTERM_BP_FAT | GO:0006928~cell motion | 7.00 | 0.94 | 0.00 | 7.76 | 779920, 825577, 823371, 819273, 785012, 799249, 810015 | 46 | 475 | 13528 | 4.333913 | 0.997817 | 0.064428 | 7.307214 |
| GOTERM_BP_FAT | GO:0045935~positive regulation of nucleobase, nucleoside, nucleotide and nucleic acid metabolic process | 7.00 | 0.94 | 0.02 | 5.92 | 803206, 783742, 825577, 781709, 819016, 781132, 818089 | 46 | 624 | 13528 | 3.299052 | 1 | 0.142991 | 23.91162 |
| GOTERM_BP_FAT | GO:0051173~positive regulation of nitrogen compound metabolic process | 7.00 | 0.94 | 0.02 | 5.71 | 803206, 783742, 825577, 781709, 819016, 781132, 818089 | 46 | 644 | 13528 | 3.196597 | 1 | 0.154978 | 27.01402 |
| GOTERM_BP_FAT | GO:0050678~regulation of epithelial cell proliferation | 6.00 | 0.81 | 0.00 | 18.09 | 823371, 781709, 819273, 799249, 817755, 798079 | 46 | 71 | 13528 | 24.85242 | 0.004722 | 2.25E-04 | 0.005861 |
| GOTERM_BP_FAT | GO:0007160~cell-matrix adhesion | 6.00 | 0.81 | 0.00 | 16.48 | 799969, 814431, 795569, 787106, 781132, 810626 | 46 | 89 | 13528 | 19.82609 | 0.014329 | 6.27E-04 | 0.017873 |
| GOTERM_BP_FAT | GO:0030198~extracellular matrix organization | 6.00 | 0.81 | 0.00 | 15.39 | 823371, 795569, 781978, 810626, 808600, 804645 | 46 | 104 | 13528 | 16.96656 | 0.030346 | 9.94E-04 | 0.038157 |
| GOTERM_BP_FAT | GO:0007568~aging | 6.00 | 0.81 | 0.00 | 15.00 | 779920, 781362, 799969, 796239, 779314, 818089 | 46 | 110 | 13528 | 16.04111 | 0.039605 | 0.001224 | 0.050033 |
| GOTERM_BP_FAT | GO:0014070~response to organic cyclic substance | 6.00 | 0.81 | 0.00 | 14.34 | 779920, 799969, 810015, 784852, 781132, 818089 | 46 | 121 | 13528 | 14.58282 | 0.061883 | 0.001877 | 0.079081 |
| GOTERM_BP_FAT | GO:0043434~response to peptide hormone stimulus | 6.00 | 0.81 | 0.00 | 12.69 | 799969, 783742, 796732, 779314, 818089, 808600 | 46 | 154 | 13528 | 11.45793 | 0.181489 | 0.004757 | 0.247714 |
| GOTERM_BP_FAT | GO:0010740~positive regulation of protein kinase cascade | 6.00 | 0.81 | 0.00 | 12.14 | 814431, 825577, 819273, 784852, 781132, 818089 | 46 | 167 | 13528 | 10.566 | 0.253508 | 0.006623 | 0.361431 |
| GOTERM_BP_FAT | GO:0030334~regulation of cell migration | 6.00 | 0.81 | 0.00 | 12.06 | 779920, 799969, 825577, 781709, 826660, 799249 | 46 | 169 | 13528 | 10.44096 | 0.26582 | 0.006843 | 0.381951 |
| GOTERM_BP_FAT | GO:0040012~regulation of locomotion | 6.00 | 0.81 | 0.00 | 11.21 | 779920, 799969, 825577, 781709, 826660, 799249 | 46 | 192 | 13528 | 9.190217 | 0.42689 | 0.011072 | 0.687046 |
| GOTERM_BP_FAT | GO:0051270~regulation of cell motion | 6.00 | 0.81 | 0.00 | 11.18 | 779920, 799969, 825577, 781709, 826660, 799249 | 46 | 193 | 13528 | 9.1426 | 0.43452 | 0.011116 | 0.703529 |
| GOTERM_BP_FAT | GO:0010035~response to inorganic substance | 6.00 | 0.81 | 0.00 | 10.78 | 779920, 799969, 810015, 781132, 818089, 808600 | 46 | 205 | 13528 | 8.607423 | 0.527954 | 0.014064 | 0.92537 |
| GOTERM_BP_FAT | GO:0010608~posttranscriptional regulation of gene expression | 6.00 | 0.81 | 0.00 | 10.59 | 779920, 781362, 799969, 825577, 781709, 797559 | 46 | 211 | 13528 | 8.362662 | 0.575006 | 0.015164 | 1.054121 |
| GOTERM_BP_FAT | GO:0045597~positive regulation of cell differentiation | 6.00 | 0.81 | 0.00 | 10.06 | 799969, 814431, 783742, 825577, 781132, 818089 | 46 | 229 | 13528 | 7.705335 | 0.709887 | 0.020082 | 1.520879 |
| GOTERM_BP_FAT | GO:0031175~neuron projection development | 6.00 | 0.81 | 0.00 | 9.35 | 779920, 799969, 825577, 819273, 785012, 815476 | 46 | 256 | 13528 | 6.892663 | 0.868895 | 0.029437 | 2.484837 |
| GOTERM_BP_FAT | GO:0051094~positive regulation of developmental process | 6.00 | 0.81 | 0.00 | 8.82 | 799969, 814431, 783742, 825577, 781132, 818089 | 46 | 278 | 13528 | 6.347201 | 0.945894 | 0.03865 | 3.54785 |
| GOTERM_BP_FAT | GO:0030030~cell projection organization | 6.00 | 0.81 | 0.01 | 7.11 | 779920, 799969, 825577, 819273, 785012, 815476 | 46 | 368 | 13528 | 4.794896 | 0.999933 | 0.082968 | 11.22496 |
| GOTERM_BP_FAT | GO:0007243~protein kinase cascade | 6.00 | 0.81 | 0.01 | 7.08 | 783742, 819273, 817755, 815476, 779314, 819639 | 46 | 370 | 13528 | 4.768978 | 0.999946 | 0.083331 | 11.46436 |
| GOTERM_BP_FAT | GO:0000278~mitotic cell cycle | 6.00 | 0.81 | 0.01 | 7.08 | 803206, 781362, 799969, 798079, 798174, 797559 | 46 | 370 | 13528 | 4.768978 | 0.999946 | 0.083331 | 11.46436 |
| GOTERM_BP_FAT | GO:0008283~cell proliferation | 6.00 | 0.81 | 0.01 | 6.12 | 779920, 799969, 816636, 781709, 819273, 781132 | 46 | 436 | 13528 | 4.047068 | 1 | 0.13045 | 21.11347 |
| GOTERM_BP_FAT | GO:0042325~regulation of phosphorylation | 6.00 | 0.81 | 0.02 | 5.74 | 779920, 781362, 799969, 825577, 819273, 822012 | 46 | 466 | 13528 | 3.786527 | 1 | 0.153012 | 26.54531 |
| GOTERM_BP_FAT | GO:0019220~regulation of phosphate metabolic process | 6.00 | 0.81 | 0.02 | 5.52 | 779920, 781362, 799969, 825577, 819273, 822012 | 46 | 485 | 13528 | 3.638189 | 1 | 0.168266 | 30.26835 |
| GOTERM_BP_FAT | GO:0051174~regulation of phosphorus metabolic process | 6.00 | 0.81 | 0.02 | 5.52 | 779920, 781362, 799969, 825577, 819273, 822012 | 46 | 485 | 13528 | 3.638189 | 1 | 0.168266 | 30.26835 |
| GOTERM_BP_FAT | GO:0048609~reproductive process in a multicellular organism | 6.00 | 0.81 | 0.02 | 5.50 | 799969, 783742, 781709, 819273, 810015, 798174 | 46 | 487 | 13528 | 3.623248 | 1 | 0.168783 | 30.67141 |
| GOTERM_BP_FAT | GO:0032504~multicellular organism reproduction | 6.00 | 0.81 | 0.02 | 5.50 | 799969, 783742, 781709, 819273, 810015, 798174 | 46 | 487 | 13528 | 3.623248 | 1 | 0.168783 | 30.67141 |
| GOTERM_BP_FAT | GO:0070271~protein complex biogenesis | 6.00 | 0.81 | 0.03 | 5.30 | 823371, 819016, 784852, 822268, 779314, 781132 | 46 | 505 | 13528 | 3.494102 | 1 | 0.18734 | 34.38247 |
| GOTERM_BP_FAT | GO:0006461~protein complex assembly | 6.00 | 0.81 | 0.03 | 5.30 | 823371, 819016, 784852, 822268, 779314, 781132 | 46 | 505 | 13528 | 3.494102 | 1 | 0.18734 | 34.38247 |
| GOTERM_BP_FAT | GO:0045941~positive regulation of transcription | 6.00 | 0.81 | 0.04 | 4.71 | 803206, 783742, 825577, 781709, 781132, 818089 | 46 | 564 | 13528 | 3.128585 | 1 | 0.242424 | 47.24589 |
| GOTERM_BP_FAT | GO:0010628~positive regulation of gene expression | 6.00 | 0.81 | 0.04 | 4.55 | 803206, 783742, 825577, 781709, 781132, 818089 | 46 | 581 | 13528 | 3.037043 | 1 | 0.259322 | 51.02194 |
| GOTERM_BP_FAT | GO:0044093~positive regulation of molecular function | 6.00 | 0.81 | 0.04 | 4.51 | 781362, 799969, 825577, 819273, 818089, 822012 | 46 | 586 | 13528 | 3.011129 | 1 | 0.261755 | 52.12869 |
| GOTERM_BP_FAT | GO:0007267~cell-cell signaling | 6.00 | 0.81 | 0.05 | 4.38 | 819591, 816636, 825577, 817755, 779314, 781132 | 46 | 600 | 13528 | 2.94087 | 1 | 0.275765 | 55.20796 |
| GOTERM_BP_FAT | GO:0065003~macromolecular complex assembly | 6.00 | 0.81 | 0.07 | 3.87 | 823371, 819016, 784852, 822268, 779314, 781132 | 46 | 665 | 13528 | 2.653416 | 1 | 0.342095 | 68.69869 |
| GOTERM_BP_FAT | GO:0043933~macromolecular complex subunit organization | 6.00 | 0.81 | 0.09 | 3.55 | 823371, 819016, 784852, 822268, 779314, 781132 | 46 | 710 | 13528 | 2.485242 | 1 | 0.385521 | 76.76483 |
| GOTERM_BP_FAT | GO:0006357~regulation of transcription from RNA polymerase II promoter | 6.00 | 0.81 | 0.09 | 3.44 | 814431, 783742, 825577, 781709, 781132, 818089 | 46 | 727 | 13528 | 2.427128 | 1 | 0.401517 | 79.46539 |
| GOTERM_BP_FAT | GO:0050679~positive regulation of epithelial cell proliferation | 5.00 | 0.67 | 0.00 | 16.63 | 823371, 781709, 819273, 799249, 817755 | 46 | 41 | 13528 | 35.86426 | 0.012978 | 5.94E-04 | 0.016176 |
| GOTERM_BP_FAT | GO:0010810~regulation of cell-substrate adhesion | 5.00 | 0.67 | 0.00 | 15.96 | 779920, 781362, 799969, 798079, 808600 | 46 | 46 | 13528 | 31.96597 | 0.020581 | 7.70E-04 | 0.025751 |
| GOTERM_BP_FAT | GO:0034097~response to cytokine stimulus | 5.00 | 0.67 | 0.00 | 12.87 | 799969, 795569, 810015, 781132, 818089 | 46 | 79 | 13528 | 18.6131 | 0.16203 | 0.004302 | 0.218685 |
| GOTERM_BP_FAT | GO:0051329~interphase of mitotic cell cycle | 5.00 | 0.67 | 0.00 | 11.39 | 803206, 781362, 799969, 798079, 797559 | 46 | 103 | 13528 | 14.27607 | 0.387919 | 0.010175 | 0.606099 |
| GOTERM_BP_FAT | GO:0051325~interphase | 5.00 | 0.67 | 0.00 | 11.24 | 803206, 781362, 799969, 798079, 797559 | 46 | 106 | 13528 | 13.87203 | 0.421655 | 0.011113 | 0.675861 |
| GOTERM_BP_FAT | GO:0048511~rhythmic process | 5.00 | 0.67 | 0.00 | 10.21 | 799969, 783742, 781709, 819273, 797559 | 46 | 128 | 13528 | 11.48777 | 0.67218 | 0.018726 | 1.371734 |
| GOTERM_BP_FAT | GO:0007584~response to nutrient | 5.00 | 0.67 | 0.00 | 9.73 | 779920, 799969, 810015, 818089, 808600 | 46 | 140 | 13528 | 10.50311 | 0.789262 | 0.023671 | 1.90996 |
| GOTERM_BP_FAT | GO:0051098~regulation of binding | 5.00 | 0.67 | 0.00 | 9.26 | 781362, 799969, 825577, 806167, 818089 | 46 | 153 | 13528 | 9.610685 | 0.884797 | 0.030834 | 2.640859 |
| GOTERM_BP_FAT | GO:0051259~protein oligomerization | 5.00 | 0.67 | 0.00 | 8.58 | 819016, 784852, 822268, 779314, 781132 | 46 | 174 | 13528 | 8.450775 | 0.968403 | 0.044439 | 4.188242 |
| GOTERM_BP_FAT | GO:0048729~tissue morphogenesis | 5.00 | 0.67 | 0.00 | 8.40 | 799969, 775320, 799249, 781132, 808600 | 46 | 180 | 13528 | 8.169082 | 0.979857 | 0.046504 | 4.72092 |
| GOTERM_BP_FAT | GO:0031667~response to nutrient levels | 5.00 | 0.67 | 0.00 | 7.94 | 779920, 799969, 810015, 818089, 808600 | 46 | 197 | 13528 | 7.464136 | 0.995468 | 0.059482 | 6.464962 |
| GOTERM_BP_FAT | GO:0006916~anti-apoptosis | 5.00 | 0.67 | 0.00 | 7.71 | 799969, 783742, 781709, 819639, 818089 | 46 | 206 | 13528 | 7.138033 | 0.998209 | 0.065073 | 7.534225 |
| GOTERM_BP_FAT | GO:0007507~heart development | 5.00 | 0.67 | 0.01 | 7.49 | 779920, 819273, 795569, 775320, 781132 | 46 | 215 | 13528 | 6.839232 | 0.999361 | 0.071611 | 8.707572 |
| GOTERM_BP_FAT | GO:0042493~response to drug | 5.00 | 0.67 | 0.01 | 7.47 | 779920, 799969, 819273, 810015, 818089 | 46 | 216 | 13528 | 6.807568 | 0.999434 | 0.072046 | 8.844438 |
| GOTERM_BP_FAT | GO:0035295~tube development | 5.00 | 0.67 | 0.01 | 7.38 | 799969, 781709, 775320, 817755, 781132 | 46 | 220 | 13528 | 6.683794 | 0.999656 | 0.075211 | 9.404954 |
| GOTERM_BP_FAT | GO:0009991~response to extracellular stimulus | 5.00 | 0.67 | 0.01 | 7.38 | 779920, 799969, 810015, 818089, 808600 | 46 | 220 | 13528 | 6.683794 | 0.999656 | 0.075211 | 9.404954 |
| GOTERM_BP_FAT | GO:0060429~epithelium development | 5.00 | 0.67 | 0.01 | 7.22 | 799969, 781709, 775320, 799249, 781132 | 46 | 227 | 13528 | 6.477686 | 0.999864 | 0.079102 | 10.43624 |
| GOTERM_BP_FAT | GO:0030097~hemopoiesis | 5.00 | 0.67 | 0.01 | 7.02 | 799969, 783742, 781709, 798079, 781132 | 46 | 236 | 13528 | 6.230656 | 0.999962 | 0.084101 | 11.85644 |
| GOTERM_BP_FAT | GO:0051240~positive regulation of multicellular organismal process | 5.00 | 0.67 | 0.01 | 6.86 | 819591, 799969, 783742, 825577, 810015 | 46 | 244 | 13528 | 6.026372 | 0.999989 | 0.090912 | 13.20743 |
| GOTERM_BP_FAT | GO:0000904~cell morphogenesis involved in differentiation | 5.00 | 0.67 | 0.01 | 6.86 | 799969, 823371, 819273, 785012, 781132 | 46 | 244 | 13528 | 6.026372 | 0.999989 | 0.090912 | 13.20743 |
| GOTERM_BP_FAT | GO:0048534~hemopoietic or lymphoid organ development | 5.00 | 0.67 | 0.01 | 6.54 | 799969, 783742, 781709, 798079, 781132 | 46 | 260 | 13528 | 5.655518 | 0.999999 | 0.105197 | 16.1549 |
| GOTERM_BP_FAT | GO:0002520~immune system development | 5.00 | 0.67 | 0.01 | 6.25 | 799969, 783742, 781709, 798079, 781132 | 46 | 276 | 13528 | 5.327662 | 1 | 0.122838 | 19.4167 |
| GOTERM_BP_FAT | GO:0016477~cell migration | 5.00 | 0.67 | 0.01 | 6.25 | 779920, 825577, 823371, 785012, 799249 | 46 | 276 | 13528 | 5.327662 | 1 | 0.122838 | 19.4167 |
| GOTERM_BP_FAT | GO:0001775~cell activation | 5.00 | 0.67 | 0.01 | 6.06 | 799969, 783742, 825577, 795569, 781132 | 46 | 287 | 13528 | 5.123466 | 1 | 0.133304 | 21.82981 |
| GOTERM_BP_FAT | GO:0051301~cell division | 5.00 | 0.67 | 0.02 | 5.93 | 803206, 781362, 798079, 798174, 797559 | 46 | 295 | 13528 | 4.984525 | 1 | 0.143284 | 23.66584 |
| GOTERM_BP_FAT | GO:0007264~small GTPase mediated signal transduction | 5.00 | 0.67 | 0.02 | 5.77 | 786559, 814239, 814431, 796239, 779314 | 46 | 305 | 13528 | 4.821098 | 1 | 0.152753 | 26.04952 |
| GOTERM_BP_FAT | GO:0048870~cell motility | 5.00 | 0.67 | 0.02 | 5.74 | 779920, 825577, 823371, 785012, 799249 | 46 | 307 | 13528 | 4.78969 | 1 | 0.153906 | 26.53733 |
| GOTERM_BP_FAT | GO:0051674~localization of cell | 5.00 | 0.67 | 0.02 | 5.74 | 779920, 825577, 823371, 785012, 799249 | 46 | 307 | 13528 | 4.78969 | 1 | 0.153906 | 26.53733 |
| GOTERM_BP_FAT | GO:0006917~induction of apoptosis | 5.00 | 0.67 | 0.02 | 5.55 | 779920, 781362, 786559, 785012, 819639 | 46 | 320 | 13528 | 4.595109 | 1 | 0.167287 | 29.79001 |
| GOTERM_BP_FAT | GO:0012502~induction of programmed cell death | 5.00 | 0.67 | 0.02 | 5.53 | 779920, 781362, 786559, 785012, 819639 | 46 | 321 | 13528 | 4.580794 | 1 | 0.167878 | 30.0457 |
| GOTERM_BP_FAT | GO:0009628~response to abiotic stimulus | 5.00 | 0.67 | 0.03 | 4.90 | 799969, 796239, 795569, 818089, 808600 | 46 | 368 | 13528 | 3.995747 | 1 | 0.220915 | 42.67815 |
| GOTERM_BP_FAT | GO:0045944~positive regulation of transcription from RNA polymerase II promoter | 5.00 | 0.67 | 0.03 | 4.87 | 783742, 825577, 781709, 781132, 818089 | 46 | 371 | 13528 | 3.963436 | 1 | 0.224908 | 43.50951 |
| GOTERM_BP_FAT | GO:0007610~behavior | 5.00 | 0.67 | 0.07 | 3.84 | 779920, 799969, 825577, 796239, 810015 | 46 | 469 | 13528 | 3.135255 | 1 | 0.345642 | 69.32621 |
| GOTERM_BP_FAT | GO:0045893~positive regulation of transcription, DNA-dependent | 5.00 | 0.67 | 0.07 | 3.77 | 783742, 825577, 781709, 781132, 818089 | 46 | 477 | 13528 | 3.082673 | 1 | 0.353721 | 71.16 |
| GOTERM_BP_FAT | GO:0051254~positive regulation of RNA metabolic process | 5.00 | 0.67 | 0.07 | 3.74 | 783742, 825577, 781709, 781132, 818089 | 46 | 481 | 13528 | 3.057037 | 1 | 0.357135 | 72.05445 |
| GOTERM_BP_FAT | GO:0009611~response to wounding | 5.00 | 0.67 | 0.10 | 3.34 | 799969, 825577, 819273, 795569, 818089 | 46 | 530 | 13528 | 2.774405 | 1 | 0.419631 | 81.69593 |
| GOTERM_BP_FAT | GO:0001952~regulation of cell-matrix adhesion | 4.00 | 0.54 | 0.00 | 13.36 | 779920, 781362, 799969, 798079 | 46 | 27 | 13528 | 43.56844 | 0.118112 | 0.003391 | 0.15554 |
| GOTERM_BP_FAT | GO:0051291~protein heterooligomerization | 4.00 | 0.54 | 0.00 | 10.53 | 784852, 822268, 779314, 781132 | 46 | 52 | 13528 | 22.62207 | 0.591936 | 0.015335 | 1.103922 |
| GOTERM_BP_FAT | GO:0042129~regulation of T cell proliferation | 4.00 | 0.54 | 0.00 | 9.78 | 781362, 783742, 825577, 819273 | 46 | 62 | 13528 | 18.97335 | 0.776664 | 0.023514 | 1.839404 |
| GOTERM_BP_FAT | GO:0022602~ovulation cycle process | 4.00 | 0.54 | 0.00 | 9.78 | 799969, 783742, 781709, 819273 | 46 | 62 | 13528 | 18.97335 | 0.776664 | 0.023514 | 1.839404 |
| GOTERM_BP_FAT | GO:0042063~gliogenesis | 4.00 | 0.54 | 0.00 | 9.59 | 816636, 819273, 798079, 781132 | 46 | 65 | 13528 | 18.09766 | 0.820844 | 0.025717 | 2.106997 |
| GOTERM_BP_FAT | GO:0042698~ovulation cycle | 4.00 | 0.54 | 0.00 | 9.46 | 799969, 783742, 781709, 819273 | 46 | 67 | 13528 | 17.55743 | 0.846984 | 0.027629 | 2.298015 |
| GOTERM_BP_FAT | GO:0030278~regulation of ossification | 4.00 | 0.54 | 0.00 | 8.83 | 799969, 825577, 798079, 781132 | 46 | 78 | 13528 | 15.08138 | 0.945376 | 0.039043 | 3.536476 |
| GOTERM_BP_FAT | GO:0050670~regulation of lymphocyte proliferation | 4.00 | 0.54 | 0.00 | 8.57 | 781362, 783742, 825577, 819273 | 46 | 83 | 13528 | 14.17287 | 0.968934 | 0.044085 | 4.208332 |
| GOTERM_BP_FAT | GO:0032944~regulation of mononuclear cell proliferation | 4.00 | 0.54 | 0.00 | 8.52 | 781362, 783742, 825577, 819273 | 46 | 84 | 13528 | 14.00414 | 0.972459 | 0.045008 | 4.35109 |
| GOTERM_BP_FAT | GO:0070663~regulation of leukocyte proliferation | 4.00 | 0.54 | 0.00 | 8.52 | 781362, 783742, 825577, 819273 | 46 | 84 | 13528 | 14.00414 | 0.972459 | 0.045008 | 4.35109 |
| GOTERM_BP_FAT | GO:0031960~response to corticosteroid stimulus | 4.00 | 0.54 | 0.00 | 8.48 | 799969, 825577, 810015, 808600 | 46 | 85 | 13528 | 13.83939 | 0.975646 | 0.045937 | 4.496667 |
| GOTERM_BP_FAT | GO:0048872~homeostasis of number of cells | 4.00 | 0.54 | 0.00 | 7.81 | 799969, 783742, 825577, 781709 | 46 | 100 | 13528 | 11.76348 | 0.997204 | 0.063242 | 7.022887 |
| GOTERM_BP_FAT | GO:0002009~morphogenesis of an epithelium | 4.00 | 0.54 | 0.00 | 7.77 | 799969, 775320, 799249, 781132 | 46 | 101 | 13528 | 11.64701 | 0.997633 | 0.064281 | 7.214292 |
| GOTERM_BP_FAT | GO:0021700~developmental maturation | 4.00 | 0.54 | 0.00 | 7.77 | 781709, 798126, 810015, 781132 | 46 | 101 | 13528 | 11.64701 | 0.997633 | 0.064281 | 7.214292 |
| GOTERM_BP_FAT | GO:0051090~regulation of transcription factor activity | 4.00 | 0.54 | 0.00 | 7.70 | 781362, 825577, 806167, 818089 | 46 | 103 | 13528 | 11.42085 | 0.998317 | 0.065026 | 7.60573 |
| GOTERM_BP_FAT | GO:0007265~Ras protein signal transduction | 4.00 | 0.54 | 0.01 | 7.62 | 814239, 814431, 796239, 779314 | 46 | 105 | 13528 | 11.20331 | 0.998818 | 0.067804 | 8.008651 |
| GOTERM_BP_FAT | GO:0043627~response to estrogen stimulus | 4.00 | 0.54 | 0.01 | 7.62 | 779920, 799969, 810015, 781132 | 46 | 105 | 13528 | 11.20331 | 0.998818 | 0.067804 | 8.008651 |
| GOTERM_BP_FAT | GO:0043408~regulation of MAPKKK cascade | 4.00 | 0.54 | 0.01 | 7.47 | 825577, 819273, 796732, 781132 | 46 | 109 | 13528 | 10.79218 | 0.999436 | 0.071394 | 8.848805 |
| GOTERM_BP_FAT | GO:0032844~regulation of homeostatic process | 4.00 | 0.54 | 0.01 | 7.29 | 799969, 783742, 781709, 798079 | 46 | 114 | 13528 | 10.31884 | 0.999791 | 0.07825 | 9.962809 |
| GOTERM_BP_FAT | GO:0001503~ossification | 4.00 | 0.54 | 0.01 | 7.25 | 799969, 798126, 810015, 808600 | 46 | 115 | 13528 | 10.22911 | 0.99983 | 0.079357 | 10.19404 |
| GOTERM_BP_FAT | GO:0050863~regulation of T cell activation | 4.00 | 0.54 | 0.01 | 7.19 | 781362, 783742, 825577, 819273 | 46 | 117 | 13528 | 10.05425 | 0.999889 | 0.080148 | 10.66484 |
| GOTERM_BP_FAT | GO:0051101~regulation of DNA binding | 4.00 | 0.54 | 0.01 | 7.05 | 781362, 825577, 806167, 818089 | 46 | 121 | 13528 | 9.721883 | 0.999954 | 0.083917 | 11.6395 |
| GOTERM_BP_FAT | GO:0060348~bone development | 4.00 | 0.54 | 0.01 | 6.99 | 799969, 798126, 810015, 808600 | 46 | 123 | 13528 | 9.563803 | 0.999971 | 0.085471 | 12.14315 |
| GOTERM_BP_FAT | GO:0035239~tube morphogenesis | 4.00 | 0.54 | 0.01 | 6.86 | 799969, 781709, 775320, 781132 | 46 | 127 | 13528 | 9.262581 | 0.999989 | 0.091463 | 13.18249 |
| GOTERM_BP_FAT | GO:0010038~response to metal ion | 4.00 | 0.54 | 0.01 | 6.80 | 779920, 799969, 810015, 781132 | 46 | 129 | 13528 | 9.118975 | 0.999993 | 0.093039 | 13.71793 |
| GOTERM_BP_FAT | GO:0001666~response to hypoxia | 4.00 | 0.54 | 0.01 | 6.65 | 799969, 781709, 798126, 784852 | 46 | 134 | 13528 | 8.778715 | 0.999998 | 0.10112 | 15.10133 |
| GOTERM_BP_FAT | GO:0051051~negative regulation of transport | 4.00 | 0.54 | 0.01 | 6.62 | 779920, 799969, 810015, 796732 | 46 | 135 | 13528 | 8.713688 | 0.999999 | 0.1023 | 15.38554 |
| GOTERM_BP_FAT | GO:0070482~response to oxygen levels | 4.00 | 0.54 | 0.01 | 6.45 | 799969, 781709, 798126, 784852 | 46 | 141 | 13528 | 8.342892 | 1 | 0.110232 | 17.14164 |
| GOTERM_BP_FAT | GO:0051249~regulation of lymphocyte activation | 4.00 | 0.54 | 0.01 | 6.26 | 781362, 783742, 825577, 819273 | 46 | 148 | 13528 | 7.948296 | 1 | 0.122903 | 19.29567 |
| GOTERM_BP_FAT | GO:0001525~angiogenesis | 4.00 | 0.54 | 0.01 | 6.26 | 779920, 781709, 817755, 781132 | 46 | 148 | 13528 | 7.948296 | 1 | 0.122903 | 19.29567 |
| GOTERM_BP_FAT | GO:0007346~regulation of mitotic cell cycle | 4.00 | 0.54 | 0.01 | 6.16 | 803206, 781362, 799969, 783742 | 46 | 152 | 13528 | 7.73913 | 1 | 0.127823 | 20.57424 |
| GOTERM_BP_FAT | GO:0032101~regulation of response to external stimulus | 4.00 | 0.54 | 0.02 | 5.99 | 783742, 825577, 781709, 810015 | 46 | 159 | 13528 | 7.398414 | 1 | 0.139216 | 22.8888 |
| GOTERM_BP_FAT | GO:0006979~response to oxidative stress | 4.00 | 0.54 | 0.02 | 5.87 | 799969, 810015, 818089, 808600 | 46 | 164 | 13528 | 7.172853 | 1 | 0.145484 | 24.59734 |
| GOTERM_BP_FAT | GO:0002694~regulation of leukocyte activation | 4.00 | 0.54 | 0.02 | 5.82 | 781362, 783742, 825577, 819273 | 46 | 166 | 13528 | 7.086433 | 1 | 0.148935 | 25.29263 |
| GOTERM_BP_FAT | GO:0050865~regulation of cell activation | 4.00 | 0.54 | 0.02 | 5.62 | 781362, 783742, 825577, 819273 | 46 | 175 | 13528 | 6.721988 | 1 | 0.160324 | 28.49689 |
| GOTERM_BP_FAT | GO:0051960~regulation of nervous system development | 4.00 | 0.54 | 0.03 | 5.28 | 819591, 799969, 814431, 818089 | 46 | 192 | 13528 | 6.126812 | 1 | 0.187928 | 34.81587 |
| GOTERM_BP_FAT | GO:0009314~response to radiation | 4.00 | 0.54 | 0.03 | 5.12 | 799969, 796239, 795569, 818089 | 46 | 200 | 13528 | 5.881739 | 1 | 0.202333 | 37.8704 |
| GOTERM_BP_FAT | GO:0060284~regulation of cell development | 4.00 | 0.54 | 0.03 | 5.03 | 819591, 799969, 814431, 818089 | 46 | 205 | 13528 | 5.738282 | 1 | 0.210871 | 39.79427 |
| GOTERM_BP_FAT | GO:0008361~regulation of cell size | 4.00 | 0.54 | 0.03 | 5.02 | 781362, 799969, 815476, 797559 | 46 | 206 | 13528 | 5.710426 | 1 | 0.212149 | 40.17992 |
| GOTERM_BP_FAT | GO:0048514~blood vessel morphogenesis | 4.00 | 0.54 | 0.03 | 4.93 | 779920, 781709, 817755, 781132 | 46 | 211 | 13528 | 5.575108 | 1 | 0.219617 | 42.11061 |
| GOTERM_BP_FAT | GO:0045596~negative regulation of cell differentiation | 4.00 | 0.54 | 0.03 | 4.84 | 814431, 783742, 798079, 781132 | 46 | 216 | 13528 | 5.446055 | 1 | 0.227078 | 44.04227 |
| GOTERM_BP_FAT | GO:0007423~sensory organ development | 4.00 | 0.54 | 0.04 | 4.63 | 799969, 781709, 819273, 781132 | 46 | 229 | 13528 | 5.13689 | 1 | 0.251387 | 49.03927 |
| GOTERM_BP_FAT | GO:0048584~positive regulation of response to stimulus | 4.00 | 0.54 | 0.04 | 4.52 | 783742, 825577, 781709, 818089 | 46 | 236 | 13528 | 4.984525 | 1 | 0.261302 | 51.69633 |
| GOTERM_BP_FAT | GO:0002684~positive regulation of immune system process | 4.00 | 0.54 | 0.04 | 4.49 | 783742, 825577, 781709, 818089 | 46 | 238 | 13528 | 4.942638 | 1 | 0.26265 | 52.44919 |
| GOTERM_BP_FAT | GO:0003006~reproductive developmental process | 4.00 | 0.54 | 0.06 | 4.15 | 799969, 783742, 781709, 810015 | 46 | 262 | 13528 | 4.489877 | 1 | 0.302555 | 61.17306 |
| GOTERM_BP_FAT | GO:0032535~regulation of cellular component size | 4.00 | 0.54 | 0.06 | 4.04 | 781362, 799969, 815476, 797559 | 46 | 271 | 13528 | 4.340767 | 1 | 0.320488 | 64.25642 |
| GOTERM_BP_FAT | GO:0016337~cell-cell adhesion | 4.00 | 0.54 | 0.06 | 3.97 | 799969, 781978, 822268, 781132 | 46 | 276 | 13528 | 4.26213 | 1 | 0.328791 | 65.91628 |
| GOTERM_BP_FAT | GO:0044057~regulation of system process | 4.00 | 0.54 | 0.08 | 3.59 | 819591, 796239, 810015, 822012 | 46 | 309 | 13528 | 3.806951 | 1 | 0.379474 | 75.78832 |
| GOTERM_BP_FAT | GO:0010812~negative regulation of cell-substrate adhesion | 3.00 | 0.40 | 0.00 | 10.74 | 779920, 781362, 808600 | 46 | 11 | 13528 | 80.20553 | 0.537682 | 0.014185 | 0.950915 |
| GOTERM_BP_FAT | GO:0051607~defense response to virus | 3.00 | 0.40 | 0.00 | 8.98 | 799969, 825577, 818089 | 46 | 20 | 13528 | 44.11304 | 0.92696 | 0.035691 | 3.188753 |
| GOTERM_BP_FAT | GO:0033077~T cell differentiation in the thymus | 3.00 | 0.40 | 0.00 | 8.12 | 799969, 783742, 781132 | 46 | 27 | 13528 | 32.67633 | 0.991494 | 0.054539 | 5.732815 |
| GOTERM_BP_FAT | GO:0032963~collagen metabolic process | 3.00 | 0.40 | 0.00 | 8.01 | 795569, 798126, 808600 | 46 | 28 | 13528 | 31.50932 | 0.994045 | 0.057191 | 6.148166 |
| GOTERM_BP_FAT | GO:0030199~collagen fibril organization | 3.00 | 0.40 | 0.00 | 7.91 | 795569, 810626, 808600 | 46 | 29 | 13528 | 30.42279 | 0.995882 | 0.059845 | 6.575722 |
| GOTERM_BP_FAT | GO:0008543~fibroblast growth factor receptor signaling pathway | 3.00 | 0.40 | 0.00 | 7.91 | 816636, 817755, 815476 | 46 | 29 | 13528 | 30.42279 | 0.995882 | 0.059845 | 6.575722 |
| GOTERM_BP_FAT | GO:0043588~skin development | 3.00 | 0.40 | 0.00 | 7.91 | 795569, 810626, 808600 | 46 | 29 | 13528 | 30.42279 | 0.995882 | 0.059845 | 6.575722 |
| GOTERM_BP_FAT | GO:0048661~positive regulation of smooth muscle cell proliferation | 3.00 | 0.40 | 0.00 | 7.72 | 825577, 781709, 810015 | 46 | 31 | 13528 | 28.46003 | 0.9981 | 0.065154 | 7.466387 |
| GOTERM_BP_FAT | GO:0044259~multicellular organismal macromolecule metabolic process | 3.00 | 0.40 | 0.00 | 7.72 | 795569, 798126, 808600 | 46 | 31 | 13528 | 28.46003 | 0.9981 | 0.065154 | 7.466387 |
| GOTERM_BP_FAT | GO:0007569~cell aging | 3.00 | 0.40 | 0.01 | 7.55 | 781362, 799969, 796239 | 46 | 33 | 13528 | 26.73518 | 0.999164 | 0.070456 | 8.402667 |
| GOTERM_BP_FAT | GO:0001776~leukocyte homeostasis | 3.00 | 0.40 | 0.01 | 7.30 | 799969, 783742, 825577 | 46 | 36 | 13528 | 24.50725 | 0.999777 | 0.078378 | 9.887857 |
| GOTERM_BP_FAT | GO:0048565~gut development | 3.00 | 0.40 | 0.01 | 7.30 | 799969, 795569, 781132 | 46 | 36 | 13528 | 24.50725 | 0.999777 | 0.078378 | 9.887857 |
| GOTERM_BP_FAT | GO:0044236~multicellular organismal metabolic process | 3.00 | 0.40 | 0.01 | 7.22 | 795569, 798126, 808600 | 46 | 37 | 13528 | 23.84489 | 0.999859 | 0.079556 | 10.40336 |
| GOTERM_BP_FAT | GO:0051781~positive regulation of cell division | 3.00 | 0.40 | 0.01 | 7.08 | 816636, 781709, 817755 | 46 | 39 | 13528 | 22.62207 | 0.999946 | 0.084036 | 11.46336 |
| GOTERM_BP_FAT | GO:0022405~hair cycle process | 3.00 | 0.40 | 0.01 | 6.87 | 799969, 810015, 818089 | 46 | 42 | 13528 | 21.00621 | 0.999988 | 0.091766 | 13.12169 |
| GOTERM_BP_FAT | GO:0022404~molting cycle process | 3.00 | 0.40 | 0.01 | 6.87 | 799969, 810015, 818089 | 46 | 42 | 13528 | 21.00621 | 0.999988 | 0.091766 | 13.12169 |
| GOTERM_BP_FAT | GO:0031647~regulation of protein stability | 3.00 | 0.40 | 0.01 | 6.87 | 779920, 781362, 799969 | 46 | 42 | 13528 | 21.00621 | 0.999988 | 0.091766 | 13.12169 |
| GOTERM_BP_FAT | GO:0001942~hair follicle development | 3.00 | 0.40 | 0.01 | 6.87 | 799969, 810015, 818089 | 46 | 42 | 13528 | 21.00621 | 0.999988 | 0.091766 | 13.12169 |
| GOTERM_BP_FAT | GO:0051591~response to cAMP | 3.00 | 0.40 | 0.01 | 6.87 | 784852, 818089, 808600 | 46 | 42 | 13528 | 21.00621 | 0.999988 | 0.091766 | 13.12169 |
| GOTERM_BP_FAT | GO:0007162~negative regulation of cell adhesion | 3.00 | 0.40 | 0.01 | 6.80 | 779920, 781362, 808600 | 46 | 43 | 13528 | 20.51769 | 0.999993 | 0.093586 | 13.69157 |
| GOTERM_BP_FAT | GO:0042303~molting cycle | 3.00 | 0.40 | 0.01 | 6.80 | 799969, 810015, 818089 | 46 | 43 | 13528 | 20.51769 | 0.999993 | 0.093586 | 13.69157 |
| GOTERM_BP_FAT | GO:0045667~regulation of osteoblast differentiation | 3.00 | 0.40 | 0.01 | 6.80 | 825577, 798079, 781132 | 46 | 43 | 13528 | 20.51769 | 0.999993 | 0.093586 | 13.69157 |
| GOTERM_BP_FAT | GO:0042633~hair cycle | 3.00 | 0.40 | 0.01 | 6.80 | 799969, 810015, 818089 | 46 | 43 | 13528 | 20.51769 | 0.999993 | 0.093586 | 13.69157 |
| GOTERM_BP_FAT | GO:0048660~regulation of smooth muscle cell proliferation | 3.00 | 0.40 | 0.01 | 6.61 | 825577, 781709, 810015 | 46 | 46 | 13528 | 19.17958 | 0.999999 | 0.10196 | 15.44863 |
| GOTERM_BP_FAT | GO:0034284~response to monosaccharide stimulus | 3.00 | 0.40 | 0.01 | 6.55 | 779920, 810015, 781978 | 46 | 47 | 13528 | 18.77151 | 0.999999 | 0.105263 | 16.0492 |
| GOTERM_BP_FAT | GO:0051302~regulation of cell division | 3.00 | 0.40 | 0.01 | 6.55 | 816636, 781709, 817755 | 46 | 47 | 13528 | 18.77151 | 0.999999 | 0.105263 | 16.0492 |
| GOTERM_BP_FAT | GO:0009746~response to hexose stimulus | 3.00 | 0.40 | 0.01 | 6.55 | 779920, 810015, 781978 | 46 | 47 | 13528 | 18.77151 | 0.999999 | 0.105263 | 16.0492 |
| GOTERM_BP_FAT | GO:0043410~positive regulation of MAPKKK cascade | 3.00 | 0.40 | 0.01 | 6.50 | 825577, 819273, 781132 | 46 | 48 | 13528 | 18.38043 | 1 | 0.107785 | 16.65674 |
| GOTERM_BP_FAT | GO:0045580~regulation of T cell differentiation | 3.00 | 0.40 | 0.01 | 6.33 | 781362, 783742, 819273 | 46 | 51 | 13528 | 17.29923 | 1 | 0.11859 | 18.51853 |
| GOTERM_BP_FAT | GO:0050864~regulation of B cell activation | 3.00 | 0.40 | 0.01 | 6.33 | 781362, 783742, 825577 | 46 | 51 | 13528 | 17.29923 | 1 | 0.11859 | 18.51853 |
| GOTERM_BP_FAT | GO:0043524~negative regulation of neuron apoptosis | 3.00 | 0.40 | 0.01 | 6.33 | 799969, 814431, 796239 | 46 | 51 | 13528 | 17.29923 | 1 | 0.11859 | 18.51853 |
| GOTERM_BP_FAT | GO:0010001~glial cell differentiation | 3.00 | 0.40 | 0.01 | 6.22 | 816636, 819273, 781132 | 46 | 53 | 13528 | 16.64643 | 1 | 0.123568 | 19.78961 |
| GOTERM_BP_FAT | GO:0001889~liver development | 3.00 | 0.40 | 0.01 | 6.22 | 819273, 781132, 818089 | 46 | 53 | 13528 | 16.64643 | 1 | 0.123568 | 19.78961 |
| GOTERM_BP_FAT | GO:0048771~tissue remodeling | 3.00 | 0.40 | 0.01 | 6.07 | 781709, 819273, 781132 | 46 | 56 | 13528 | 15.75466 | 1 | 0.133592 | 21.73571 |
| GOTERM_BP_FAT | GO:0000082~G1/S transition of mitotic cell cycle | 3.00 | 0.40 | 0.01 | 6.07 | 781362, 799969, 797559 | 46 | 56 | 13528 | 15.75466 | 1 | 0.133592 | 21.73571 |
| GOTERM_BP_FAT | GO:0042542~response to hydrogen peroxide | 3.00 | 0.40 | 0.01 | 6.07 | 799969, 818089, 808600 | 46 | 56 | 13528 | 15.75466 | 1 | 0.133592 | 21.73571 |
| GOTERM_BP_FAT | GO:0009743~response to carbohydrate stimulus | 3.00 | 0.40 | 0.02 | 5.88 | 779920, 810015, 781978 | 46 | 60 | 13528 | 14.70435 | 1 | 0.145121 | 24.39318 |
| GOTERM_BP_FAT | GO:0045785~positive regulation of cell adhesion | 3.00 | 0.40 | 0.02 | 5.88 | 819273, 817755, 798079 | 46 | 60 | 13528 | 14.70435 | 1 | 0.145121 | 24.39318 |
| GOTERM_BP_FAT | GO:0001894~tissue homeostasis | 3.00 | 0.40 | 0.02 | 5.75 | 799969, 781709, 781132 | 46 | 63 | 13528 | 14.00414 | 1 | 0.154152 | 26.42432 |
| GOTERM_BP_FAT | GO:0045619~regulation of lymphocyte differentiation | 3.00 | 0.40 | 0.02 | 5.75 | 781362, 783742, 819273 | 46 | 63 | 13528 | 14.00414 | 1 | 0.154152 | 26.42432 |
| GOTERM_BP_FAT | GO:0010243~response to organic nitrogen | 3.00 | 0.40 | 0.02 | 5.75 | 810015, 784852, 818089 | 46 | 63 | 13528 | 14.00414 | 1 | 0.154152 | 26.42432 |
| GOTERM_BP_FAT | GO:0045765~regulation of angiogenesis | 3.00 | 0.40 | 0.02 | 5.75 | 825577, 819273, 804645 | 46 | 63 | 13528 | 14.00414 | 1 | 0.154152 | 26.42432 |
| GOTERM_BP_FAT | GO:0008585~female gonad development | 3.00 | 0.40 | 0.02 | 5.71 | 799969, 783742, 781709 | 46 | 64 | 13528 | 13.78533 | 1 | 0.154615 | 27.10729 |
| GOTERM_BP_FAT | GO:0045471~response to ethanol | 3.00 | 0.40 | 0.02 | 5.71 | 779920, 799969, 784852 | 46 | 64 | 13528 | 13.78533 | 1 | 0.154615 | 27.10729 |
| GOTERM_BP_FAT | GO:0032103~positive regulation of response to external stimulus | 3.00 | 0.40 | 0.02 | 5.71 | 783742, 825577, 781709 | 46 | 64 | 13528 | 13.78533 | 1 | 0.154615 | 27.10729 |
| GOTERM_BP_FAT | GO:0030217~T cell differentiation | 3.00 | 0.40 | 0.02 | 5.67 | 799969, 783742, 781132 | 46 | 65 | 13528 | 13.57324 | 1 | 0.157897 | 27.79282 |
| GOTERM_BP_FAT | GO:0048754~branching morphogenesis of a tube | 3.00 | 0.40 | 0.02 | 5.67 | 799969, 781709, 781132 | 46 | 65 | 13528 | 13.57324 | 1 | 0.157897 | 27.79282 |
| GOTERM_BP_FAT | GO:0033273~response to vitamin | 3.00 | 0.40 | 0.02 | 5.62 | 799969, 810015, 818089 | 46 | 66 | 13528 | 13.36759 | 1 | 0.161177 | 28.48066 |
| GOTERM_BP_FAT | GO:0046545~development of primary female sexual characteristics | 3.00 | 0.40 | 0.02 | 5.50 | 799969, 783742, 781709 | 46 | 69 | 13528 | 12.78639 | 1 | 0.169046 | 30.55577 |
| GOTERM_BP_FAT | GO:0010720~positive regulation of cell development | 3.00 | 0.40 | 0.02 | 5.50 | 799969, 814431, 818089 | 46 | 69 | 13528 | 12.78639 | 1 | 0.169046 | 30.55577 |
| GOTERM_BP_FAT | GO:0046660~female sex differentiation | 3.00 | 0.40 | 0.02 | 5.50 | 799969, 783742, 781709 | 46 | 69 | 13528 | 12.78639 | 1 | 0.169046 | 30.55577 |
| GOTERM_BP_FAT | GO:0019221~cytokine-mediated signaling pathway | 3.00 | 0.40 | 0.02 | 5.46 | 783742, 825577, 818089 | 46 | 70 | 13528 | 12.60373 | 1 | 0.171322 | 31.25057 |
| GOTERM_BP_FAT | GO:0045637~regulation of myeloid cell differentiation | 3.00 | 0.40 | 0.02 | 5.46 | 783742, 798079, 781132 | 46 | 70 | 13528 | 12.60373 | 1 | 0.171322 | 31.25057 |
| GOTERM_BP_FAT | GO:0031349~positive regulation of defense response | 3.00 | 0.40 | 0.02 | 5.35 | 783742, 825577, 818089 | 46 | 73 | 13528 | 12.08577 | 1 | 0.183032 | 33.34116 |
| GOTERM_BP_FAT | GO:0001763~morphogenesis of a branching structure | 3.00 | 0.40 | 0.03 | 5.32 | 799969, 781709, 781132 | 46 | 74 | 13528 | 11.92244 | 1 | 0.186274 | 34.03937 |
| GOTERM_BP_FAT | GO:0042035~regulation of cytokine biosynthetic process | 3.00 | 0.40 | 0.03 | 5.32 | 783742, 825577, 818089 | 46 | 74 | 13528 | 11.92244 | 1 | 0.186274 | 34.03937 |
| GOTERM_BP_FAT | GO:0000302~response to reactive oxygen species | 3.00 | 0.40 | 0.03 | 5.28 | 799969, 818089, 808600 | 46 | 75 | 13528 | 11.76348 | 1 | 0.188478 | 34.7379 |
| GOTERM_BP_FAT | GO:0050727~regulation of inflammatory response | 3.00 | 0.40 | 0.03 | 5.24 | 783742, 825577, 810015 | 46 | 76 | 13528 | 11.6087 | 1 | 0.189648 | 35.43655 |
| GOTERM_BP_FAT | GO:0051384~response to glucocorticoid stimulus | 3.00 | 0.40 | 0.03 | 5.17 | 799969, 825577, 810015 | 46 | 78 | 13528 | 11.31104 | 1 | 0.197075 | 36.83342 |
| GOTERM_BP_FAT | GO:0010959~regulation of metal ion transport | 3.00 | 0.40 | 0.03 | 5.07 | 799969, 810015, 796732 | 46 | 81 | 13528 | 10.89211 | 1 | 0.206634 | 38.92472 |
| GOTERM_BP_FAT | GO:0045786~negative regulation of cell cycle | 3.00 | 0.40 | 0.03 | 5.07 | 781362, 799969, 798079 | 46 | 81 | 13528 | 10.89211 | 1 | 0.206634 | 38.92472 |
| GOTERM_BP_FAT | GO:0002683~negative regulation of immune system process | 3.00 | 0.40 | 0.03 | 5.01 | 781362, 819273, 795569 | 46 | 83 | 13528 | 10.62965 | 1 | 0.211892 | 40.3141 |
| GOTERM_BP_FAT | GO:0048871~multicellular organismal homeostasis | 3.00 | 0.40 | 0.03 | 4.95 | 799969, 781709, 781132 | 46 | 85 | 13528 | 10.37954 | 1 | 0.219273 | 41.69796 |
| GOTERM_BP_FAT | GO:0002237~response to molecule of bacterial origin | 3.00 | 0.40 | 0.03 | 4.92 | 825577, 810015, 818089 | 46 | 86 | 13528 | 10.25885 | 1 | 0.220223 | 42.38739 |
| GOTERM_BP_FAT | GO:0030335~positive regulation of cell migration | 3.00 | 0.40 | 0.04 | 4.82 | 799969, 825577, 781709 | 46 | 89 | 13528 | 9.913043 | 1 | 0.228445 | 44.44381 |
| GOTERM_BP_FAT | GO:0043523~regulation of neuron apoptosis | 3.00 | 0.40 | 0.04 | 4.80 | 799969, 814431, 796239 | 46 | 90 | 13528 | 9.802899 | 1 | 0.230433 | 45.1248 |
| GOTERM_BP_FAT | GO:0043123~positive regulation of I-kappaB kinase/NF-kappaB cascade | 3.00 | 0.40 | 0.04 | 4.60 | 814431, 784852, 818089 | 46 | 97 | 13528 | 9.095473 | 1 | 0.255132 | 49.8143 |
| GOTERM_BP_FAT | GO:0040017~positive regulation of locomotion | 3.00 | 0.40 | 0.04 | 4.57 | 799969, 825577, 781709 | 46 | 98 | 13528 | 9.002662 | 1 | 0.258145 | 50.47159 |
| GOTERM_BP_FAT | GO:0051272~positive regulation of cell motion | 3.00 | 0.40 | 0.04 | 4.57 | 799969, 825577, 781709 | 46 | 98 | 13528 | 9.002662 | 1 | 0.258145 | 50.47159 |
| GOTERM_BP_FAT | GO:0030324~lung development | 3.00 | 0.40 | 0.04 | 4.55 | 781709, 817755, 781132 | 46 | 99 | 13528 | 8.911726 | 1 | 0.258825 | 51.12535 |
| GOTERM_BP_FAT | GO:0043269~regulation of ion transport | 3.00 | 0.40 | 0.04 | 4.55 | 799969, 810015, 796732 | 46 | 99 | 13528 | 8.911726 | 1 | 0.258825 | 51.12535 |
| GOTERM_BP_FAT | GO:0032868~response to insulin stimulus | 3.00 | 0.40 | 0.04 | 4.52 | 796732, 779314, 818089 | 46 | 100 | 13528 | 8.822609 | 1 | 0.260656 | 51.77548 |
| GOTERM_BP_FAT | GO:0030323~respiratory tube development | 3.00 | 0.40 | 0.05 | 4.47 | 781709, 817755, 781132 | 46 | 102 | 13528 | 8.649616 | 1 | 0.265426 | 53.0644 |
| GOTERM_BP_FAT | GO:0030098~lymphocyte differentiation | 3.00 | 0.40 | 0.05 | 4.44 | 799969, 783742, 781132 | 46 | 103 | 13528 | 8.56564 | 1 | 0.26722 | 53.70298 |
| GOTERM_BP_FAT | GO:0060249~anatomical structure homeostasis | 3.00 | 0.40 | 0.05 | 4.37 | 799969, 781709, 781132 | 46 | 106 | 13528 | 8.323216 | 1 | 0.277113 | 55.59406 |
| GOTERM_BP_FAT | GO:0043122~regulation of I-kappaB kinase/NF-kappaB cascade | 3.00 | 0.40 | 0.05 | 4.34 | 814431, 784852, 818089 | 46 | 107 | 13528 | 8.245429 | 1 | 0.278846 | 56.21589 |
| GOTERM_BP_FAT | GO:0060541~respiratory system development | 3.00 | 0.40 | 0.05 | 4.32 | 781709, 817755, 781132 | 46 | 108 | 13528 | 8.169082 | 1 | 0.281725 | 56.83332 |
| GOTERM_BP_FAT | GO:0009615~response to virus | 3.00 | 0.40 | 0.05 | 4.30 | 799969, 825577, 818089 | 46 | 109 | 13528 | 8.094136 | 1 | 0.284592 | 57.44627 |
| GOTERM_BP_FAT | GO:0007565~female pregnancy | 3.00 | 0.40 | 0.05 | 4.27 | 799969, 783742, 810015 | 46 | 110 | 13528 | 8.020553 | 1 | 0.287447 | 58.05466 |
| GOTERM_BP_FAT | GO:0007611~learning or memory | 3.00 | 0.40 | 0.05 | 4.25 | 779920, 796239, 810015 | 46 | 111 | 13528 | 7.948296 | 1 | 0.289126 | 58.65842 |
| GOTERM_BP_FAT | GO:0008406~gonad development | 3.00 | 0.40 | 0.05 | 4.23 | 799969, 783742, 781709 | 46 | 112 | 13528 | 7.877329 | 1 | 0.291954 | 59.25747 |
| GOTERM_BP_FAT | GO:0014706~striated muscle tissue development | 3.00 | 0.40 | 0.06 | 4.07 | 779920, 814431, 819273 | 46 | 119 | 13528 | 7.413957 | 1 | 0.31502 | 63.31404 |
| GOTERM_BP_FAT | GO:0042692~muscle cell differentiation | 3.00 | 0.40 | 0.06 | 4.03 | 796239, 819273, 781132 | 46 | 121 | 13528 | 7.291412 | 1 | 0.320491 | 64.4275 |
| GOTERM_BP_FAT | GO:0060537~muscle tissue development | 3.00 | 0.40 | 0.06 | 3.95 | 779920, 814431, 819273 | 46 | 125 | 13528 | 7.058087 | 1 | 0.331283 | 66.59148 |
| GOTERM_BP_FAT | GO:0042110~T cell activation | 3.00 | 0.40 | 0.07 | 3.93 | 799969, 783742, 781132 | 46 | 126 | 13528 | 7.00207 | 1 | 0.33395 | 67.11918 |
| GOTERM_BP_FAT | GO:0048608~reproductive structure development | 3.00 | 0.40 | 0.07 | 3.93 | 799969, 783742, 781709 | 46 | 126 | 13528 | 7.00207 | 1 | 0.33395 | 67.11918 |
| GOTERM_BP_FAT | GO:0045137~development of primary sexual characteristics | 3.00 | 0.40 | 0.07 | 3.91 | 799969, 783742, 781709 | 46 | 127 | 13528 | 6.946936 | 1 | 0.336605 | 67.6415 |
| GOTERM_BP_FAT | GO:0002521~leukocyte differentiation | 3.00 | 0.40 | 0.07 | 3.83 | 799969, 783742, 781132 | 46 | 131 | 13528 | 6.734816 | 1 | 0.347099 | 69.67663 |
| GOTERM_BP_FAT | GO:0016055~Wnt receptor signaling pathway | 3.00 | 0.40 | 0.07 | 3.79 | 775320, 804749, 781132 | 46 | 133 | 13528 | 6.63354 | 1 | 0.352271 | 70.66149 |
| GOTERM_BP_FAT | GO:0032870~cellular response to hormone stimulus | 3.00 | 0.40 | 0.07 | 3.79 | 783742, 796732, 779314 | 46 | 133 | 13528 | 6.63354 | 1 | 0.352271 | 70.66149 |
| GOTERM_BP_FAT | GO:0002252~immune effector process | 3.00 | 0.40 | 0.07 | 3.77 | 799969, 825577, 818089 | 46 | 134 | 13528 | 6.584036 | 1 | 0.354839 | 71.1457 |
| GOTERM_BP_FAT | GO:0048732~gland development | 3.00 | 0.40 | 0.07 | 3.75 | 799969, 783742, 819273 | 46 | 135 | 13528 | 6.535266 | 1 | 0.354939 | 71.62443 |
| GOTERM_BP_FAT | GO:0050804~regulation of synaptic transmission | 3.00 | 0.40 | 0.08 | 3.74 | 819591, 796239, 810015 | 46 | 136 | 13528 | 6.487212 | 1 | 0.356264 | 72.09768 |
| GOTERM_BP_FAT | GO:0009416~response to light stimulus | 3.00 | 0.40 | 0.08 | 3.70 | 799969, 796239, 818089 | 46 | 138 | 13528 | 6.393195 | 1 | 0.361308 | 73.02769 |
| GOTERM_BP_FAT | GO:0032880~regulation of protein localization | 3.00 | 0.40 | 0.08 | 3.70 | 799969, 814431, 825577 | 46 | 138 | 13528 | 6.393195 | 1 | 0.361308 | 73.02769 |
| GOTERM_BP_FAT | GO:0045165~cell fate commitment | 3.00 | 0.40 | 0.08 | 3.68 | 799969, 817755, 781132 | 46 | 139 | 13528 | 6.347201 | 1 | 0.363811 | 73.48447 |
| GOTERM_BP_FAT | GO:0044087~regulation of cellular component biogenesis | 3.00 | 0.40 | 0.08 | 3.63 | 779920, 819591, 814431 | 46 | 142 | 13528 | 6.213105 | 1 | 0.372469 | 74.82195 |
| GOTERM_BP_FAT | GO:0051969~regulation of transmission of nerve impulse | 3.00 | 0.40 | 0.09 | 3.54 | 819591, 796239, 810015 | 46 | 147 | 13528 | 6.001775 | 1 | 0.385854 | 76.94213 |
| GOTERM_BP_FAT | GO:0007548~sex differentiation | 3.00 | 0.40 | 0.09 | 3.48 | 799969, 783742, 781709 | 46 | 151 | 13528 | 5.842787 | 1 | 0.396597 | 78.54136 |
| GOTERM_BP_FAT | GO:0031644~regulation of neurological system process | 3.00 | 0.40 | 0.09 | 3.45 | 819591, 796239, 810015 | 46 | 153 | 13528 | 5.766411 | 1 | 0.402521 | 79.30912 |
| GOTERM_BP_FAT | GO:0033088~negative regulation of immature T cell proliferation in the thymus | 2.00 | 0.27 | 0.01 | 7.23 | 781362, 819273 | 46 | 2 | 13528 | 294.087 | 0.99985 | 0.079696 | 10.32927 |
| GOTERM_BP_FAT | GO:0060346~bone trabecula formation | 2.00 | 0.27 | 0.01 | 7.23 | 798126, 808600 | 46 | 2 | 13528 | 294.087 | 0.99985 | 0.079696 | 10.32927 |
| GOTERM_BP_FAT | GO:0033084~regulation of immature T cell proliferation in the thymus | 2.00 | 0.27 | 0.01 | 7.23 | 781362, 819273 | 46 | 2 | 13528 | 294.087 | 0.99985 | 0.079696 | 10.32927 |
| GOTERM_BP_FAT | GO:0033087~negative regulation of immature T cell proliferation | 2.00 | 0.27 | 0.01 | 7.23 | 781362, 819273 | 46 | 2 | 13528 | 294.087 | 0.99985 | 0.079696 | 10.32927 |
| GOTERM_BP_FAT | GO:0033083~regulation of immature T cell proliferation | 2.00 | 0.27 | 0.01 | 6.65 | 781362, 819273 | 46 | 3 | 13528 | 196.058 | 0.999998 | 0.1018 | 15.08714 |
| GOTERM_BP_FAT | GO:0033033~negative regulation of myeloid cell apoptosis | 2.00 | 0.27 | 0.01 | 6.24 | 799969, 783742 | 46 | 4 | 13528 | 147.0435 | 1 | 0.123137 | 19.5929 |
| GOTERM_BP_FAT | GO:0001957~intramembranous ossification | 2.00 | 0.27 | 0.02 | 5.92 | 798126, 808600 | 46 | 5 | 13528 | 117.6348 | 1 | 0.143591 | 23.85986 |
| GOTERM_BP_FAT | GO:0032964~collagen biosynthetic process | 2.00 | 0.27 | 0.02 | 5.92 | 795569, 808600 | 46 | 5 | 13528 | 117.6348 | 1 | 0.143591 | 23.85986 |
| GOTERM_BP_FAT | GO:0060343~trabecula formation | 2.00 | 0.27 | 0.03 | 5.25 | 798126, 808600 | 46 | 8 | 13528 | 73.52174 | 1 | 0.190151 | 35.35115 |
| GOTERM_BP_FAT | GO:0010224~response to UV-B | 2.00 | 0.27 | 0.03 | 5.25 | 799969, 818089 | 46 | 8 | 13528 | 73.52174 | 1 | 0.190151 | 35.35115 |
| GOTERM_BP_FAT | GO:0033032~regulation of myeloid cell apoptosis | 2.00 | 0.27 | 0.03 | 5.25 | 799969, 783742 | 46 | 8 | 13528 | 73.52174 | 1 | 0.190151 | 35.35115 |
| GOTERM_BP_FAT | GO:0060325~face morphogenesis | 2.00 | 0.27 | 0.03 | 5.08 | 798126, 808600 | 46 | 9 | 13528 | 65.35266 | 1 | 0.206839 | 38.78287 |
| GOTERM_BP_FAT | GO:0001953~negative regulation of cell-matrix adhesion | 2.00 | 0.27 | 0.03 | 4.93 | 779920, 781362 | 46 | 10 | 13528 | 58.81739 | 1 | 0.220231 | 42.03265 |
| GOTERM_BP_FAT | GO:0033138~positive regulation of peptidyl-serine phosphorylation | 2.00 | 0.27 | 0.03 | 4.93 | 799969, 825577 | 46 | 10 | 13528 | 58.81739 | 1 | 0.220231 | 42.03265 |
| GOTERM_BP_FAT | GO:0060323~head morphogenesis | 2.00 | 0.27 | 0.04 | 4.80 | 798126, 808600 | 46 | 11 | 13528 | 53.47036 | 1 | 0.231437 | 45.11014 |
| GOTERM_BP_FAT | GO:0060324~face development | 2.00 | 0.27 | 0.04 | 4.80 | 798126, 808600 | 46 | 11 | 13528 | 53.47036 | 1 | 0.231437 | 45.11014 |
| GOTERM_BP_FAT | GO:0010559~regulation of glycoprotein biosynthetic process | 2.00 | 0.27 | 0.04 | 4.80 | 799969, 781132 | 46 | 11 | 13528 | 53.47036 | 1 | 0.231437 | 45.11014 |
| GOTERM_BP_FAT | GO:0033081~regulation of T cell differentiation in the thymus | 2.00 | 0.27 | 0.04 | 4.80 | 781362, 819273 | 46 | 11 | 13528 | 53.47036 | 1 | 0.231437 | 45.11014 |
| GOTERM_BP_FAT | GO:0051926~negative regulation of calcium ion transport | 2.00 | 0.27 | 0.04 | 4.67 | 799969, 810015 | 46 | 12 | 13528 | 49.01449 | 1 | 0.246154 | 48.02445 |
| GOTERM_BP_FAT | GO:0045995~regulation of embryonic development | 2.00 | 0.27 | 0.04 | 4.56 | 826660, 799249 | 46 | 13 | 13528 | 45.24415 | 1 | 0.25897 | 50.78423 |
| GOTERM_BP_FAT | GO:0032330~regulation of chondrocyte differentiation | 2.00 | 0.27 | 0.05 | 4.45 | 781132, 818089 | 46 | 14 | 13528 | 42.01242 | 1 | 0.26641 | 53.39766 |
| GOTERM_BP_FAT | GO:0060322~head development | 2.00 | 0.27 | 0.05 | 4.45 | 798126, 808600 | 46 | 14 | 13528 | 42.01242 | 1 | 0.26641 | 53.39766 |
| GOTERM_BP_FAT | GO:0033135~regulation of peptidyl-serine phosphorylation | 2.00 | 0.27 | 0.05 | 4.45 | 799969, 825577 | 46 | 14 | 13528 | 42.01242 | 1 | 0.26641 | 53.39766 |
| GOTERM_BP_FAT | GO:0010171~body morphogenesis | 2.00 | 0.27 | 0.05 | 4.45 | 798126, 808600 | 46 | 14 | 13528 | 42.01242 | 1 | 0.26641 | 53.39766 |
| GOTERM_BP_FAT | GO:0002687~positive regulation of leukocyte migration | 2.00 | 0.27 | 0.05 | 4.36 | 825577, 781709 | 46 | 15 | 13528 | 39.21159 | 1 | 0.277766 | 55.8725 |
| GOTERM_BP_FAT | GO:0022612~gland morphogenesis | 2.00 | 0.27 | 0.05 | 4.27 | 799969, 819273 | 46 | 16 | 13528 | 36.76087 | 1 | 0.287353 | 58.21607 |
| GOTERM_BP_FAT | GO:0046579~positive regulation of Ras protein signal transduction | 2.00 | 0.27 | 0.05 | 4.27 | 796239, 819273 | 46 | 16 | 13528 | 36.76087 | 1 | 0.287353 | 58.21607 |
| GOTERM_BP_FAT | GO:0051057~positive regulation of small GTPase mediated signal transduction | 2.00 | 0.27 | 0.06 | 4.18 | 796239, 819273 | 46 | 17 | 13528 | 34.59847 | 1 | 0.298712 | 60.43534 |
| GOTERM_BP_FAT | GO:0007492~endoderm development | 2.00 | 0.27 | 0.06 | 4.10 | 823371, 781132 | 46 | 18 | 13528 | 32.67633 | 1 | 0.310781 | 62.53689 |
| GOTERM_BP_FAT | GO:0045646~regulation of erythrocyte differentiation | 2.00 | 0.27 | 0.06 | 4.10 | 783742, 798079 | 46 | 18 | 13528 | 32.67633 | 1 | 0.310781 | 62.53689 |
| GOTERM_BP_FAT | GO:0000080~G1 phase of mitotic cell cycle | 2.00 | 0.27 | 0.06 | 4.10 | 803206, 798079 | 46 | 18 | 13528 | 32.67633 | 1 | 0.310781 | 62.53689 |
| GOTERM_BP_FAT | GO:0048008~platelet-derived growth factor receptor signaling pathway | 2.00 | 0.27 | 0.06 | 4.03 | 779920, 781709 | 46 | 19 | 13528 | 30.95652 | 1 | 0.319989 | 64.52696 |
| GOTERM_BP_FAT | GO:0048678~response to axon injury | 2.00 | 0.27 | 0.06 | 3.95 | 799969, 819273 | 46 | 20 | 13528 | 29.4087 | 1 | 0.331187 | 66.41144 |
| GOTERM_BP_FAT | GO:0032570~response to progesterone stimulus | 2.00 | 0.27 | 0.06 | 3.95 | 819273, 818089 | 46 | 20 | 13528 | 29.4087 | 1 | 0.331187 | 66.41144 |
| GOTERM_BP_FAT | GO:0002685~regulation of leukocyte migration | 2.00 | 0.27 | 0.06 | 3.95 | 825577, 781709 | 46 | 20 | 13528 | 29.4087 | 1 | 0.331187 | 66.41144 |
| GOTERM_BP_FAT | GO:0032845~negative regulation of homeostatic process | 2.00 | 0.27 | 0.07 | 3.89 | 799969, 781709 | 46 | 21 | 13528 | 28.00828 | 1 | 0.339529 | 68.19595 |
| GOTERM_BP_FAT | GO:0001569~patterning of blood vessels | 2.00 | 0.27 | 0.07 | 3.89 | 781709, 781132 | 46 | 21 | 13528 | 28.00828 | 1 | 0.339529 | 68.19595 |
| GOTERM_BP_FAT | GO:0051318~G1 phase | 2.00 | 0.27 | 0.07 | 3.89 | 803206, 798079 | 46 | 21 | 13528 | 28.00828 | 1 | 0.339529 | 68.19595 |
| GOTERM_BP_FAT | GO:0043029~T cell homeostasis | 2.00 | 0.27 | 0.07 | 3.89 | 799969, 783742 | 46 | 21 | 13528 | 28.00828 | 1 | 0.339529 | 68.19595 |
| GOTERM_BP_FAT | GO:0048538~thymus development | 2.00 | 0.27 | 0.07 | 3.82 | 799969, 781132 | 46 | 22 | 13528 | 26.73518 | 1 | 0.34748 | 69.88576 |
| GOTERM_BP_FAT | GO:0007044~cell-substrate junction assembly | 2.00 | 0.27 | 0.07 | 3.76 | 799969, 823371 | 46 | 23 | 13528 | 25.57278 | 1 | 0.355065 | 71.48591 |
| GOTERM_BP_FAT | GO:0043271~negative regulation of ion transport | 2.00 | 0.27 | 0.07 | 3.76 | 799969, 810015 | 46 | 23 | 13528 | 25.57278 | 1 | 0.355065 | 71.48591 |
| GOTERM_BP_FAT | GO:0045727~positive regulation of translation | 2.00 | 0.27 | 0.07 | 3.76 | 825577, 797559 | 46 | 23 | 13528 | 25.57278 | 1 | 0.355065 | 71.48591 |
| GOTERM_BP_FAT | GO:0050873~brown fat cell differentiation | 2.00 | 0.27 | 0.08 | 3.70 | 826660, 822774 | 46 | 24 | 13528 | 24.50725 | 1 | 0.36231 | 73.00115 |
| GOTERM_BP_FAT | GO:0001101~response to acid | 2.00 | 0.27 | 0.08 | 3.70 | 799969, 818089 | 46 | 24 | 13528 | 24.50725 | 1 | 0.36231 | 73.00115 |
| GOTERM_BP_FAT | GO:0040018~positive regulation of multicellular organism growth | 2.00 | 0.27 | 0.08 | 3.64 | 799969, 783742 | 46 | 25 | 13528 | 23.52696 | 1 | 0.370455 | 74.43597 |
| GOTERM_BP_FAT | GO:0048730~epidermis morphogenesis | 2.00 | 0.27 | 0.08 | 3.64 | 799969, 808600 | 46 | 25 | 13528 | 23.52696 | 1 | 0.370455 | 74.43597 |
| GOTERM_BP_FAT | GO:0048742~regulation of skeletal muscle fiber development | 2.00 | 0.27 | 0.08 | 3.64 | 819591, 799969 | 46 | 25 | 13528 | 23.52696 | 1 | 0.370455 | 74.43597 |
| GOTERM_BP_FAT | GO:0045669~positive regulation of osteoblast differentiation | 2.00 | 0.27 | 0.08 | 3.64 | 825577, 781132 | 46 | 25 | 13528 | 23.52696 | 1 | 0.370455 | 74.43597 |
| GOTERM_BP_FAT | GO:0048146~positive regulation of fibroblast proliferation | 2.00 | 0.27 | 0.08 | 3.59 | 798079, 797559 | 46 | 26 | 13528 | 22.62207 | 1 | 0.378299 | 75.79463 |
| GOTERM_BP_FAT | GO:0042130~negative regulation of T cell proliferation | 2.00 | 0.27 | 0.09 | 3.54 | 781362, 819273 | 46 | 27 | 13528 | 21.78422 | 1 | 0.385856 | 77.08118 |
| GOTERM_BP_FAT | GO:0002260~lymphocyte homeostasis | 2.00 | 0.27 | 0.09 | 3.49 | 799969, 783742 | 46 | 28 | 13528 | 21.00621 | 1 | 0.395611 | 78.29943 |
| GOTERM_BP_FAT | GO:0048641~regulation of skeletal muscle tissue development | 2.00 | 0.27 | 0.09 | 3.44 | 819591, 799969 | 46 | 29 | 13528 | 20.28186 | 1 | 0.402637 | 79.45301 |
| GOTERM_BP_FAT | GO:0050921~positive regulation of chemotaxis | 2.00 | 0.27 | 0.09 | 3.44 | 825577, 781709 | 46 | 29 | 13528 | 20.28186 | 1 | 0.402637 | 79.45301 |
| GOTERM_BP_FAT | GO:0030856~regulation of epithelial cell differentiation | 2.00 | 0.27 | 0.09 | 3.44 | 783742, 781132 | 46 | 29 | 13528 | 20.28186 | 1 | 0.402637 | 79.45301 |
| GOTERM_BP_FAT | GO:0045638~negative regulation of myeloid cell differentiation | 2.00 | 0.27 | 0.10 | 3.39 | 783742, 781132 | 46 | 30 | 13528 | 19.6058 | 1 | 0.410661 | 80.54535 |
| GOTERM_BP_FAT | GO:0050729~positive regulation of inflammatory response | 2.00 | 0.27 | 0.10 | 3.39 | 783742, 825577 | 46 | 30 | 13528 | 19.6058 | 1 | 0.410661 | 80.54535 |
| GOTERM_BP_FAT | GO:0050920~regulation of chemotaxis | 2.00 | 0.27 | 0.10 | 3.35 | 825577, 781709 | 46 | 31 | 13528 | 18.97335 | 1 | 0.419707 | 81.57969 |
| GOTERM_BP_FAT | GO:0008637~apoptotic mitochondrial changes | 2.00 | 0.27 | 0.10 | 3.35 | 781362, 799969 | 46 | 31 | 13528 | 18.97335 | 1 | 0.419707 | 81.57969 |

| OFFICIAL_GENE_SYMBOL | Name | Species |
| --- | --- | --- |
| 817755 | fibroblast growth factor 1 (acidic) | Homo sapiens |
| 787106 | integrin, alpha 3 (antigen CD49C, alpha 3 subunit of VLA-3 receptor) | Homo sapiens |
| 779920 | phosphatase and tensin homolog; phosphatase and tensin homolog pseudogene 1 | Homo sapiens |
| 795569 | collagen, type III, alpha 1 | Homo sapiens |
| 816636 | fibroblast growth factor 5 | Homo sapiens |
| 822012 | syndecan 4 | Homo sapiens |
| 814239 | Ras association (RalGDS/AF-6) domain family member 1 | Homo sapiens |
| 819273 | v-erb-b2 erythroblastic leukemia viral oncogene homolog 2, neuro/glioblastoma derived oncogene homolog (avian) | Homo sapiens |
| 819016 | RAD51 homolog (RecA homolog, E. coli) (S. cerevisiae) | Homo sapiens |
| 826660 | laminin, alpha 4 | Homo sapiens |
| 799969 | B-cell CLL/lymphoma 2 | Homo sapiens |
| 815476 | fibroblast growth factor receptor 1 | Homo sapiens |
| 779314 | growth factor receptor-bound protein 2 | Homo sapiens |
| 784852 | baculoviral IAP repeat-containing 2 | Homo sapiens |
| 775320 | dishevelled, dsh homolog 2 (Drosophila) | Homo sapiens |
| 800275 | collagen, type IV, alpha 1 | Homo sapiens |
| 804645 | collagen, type IV, alpha 2 | Homo sapiens |
| 822268 | junction plakoglobin | Homo sapiens |
| 825577 | interleukin 6 (interferon, beta 2) | Homo sapiens |
| 785012 | deleted in colorectal carcinoma | Homo sapiens |
| 803206 | cyclin-dependent kinase 2 | Homo sapiens |
| 808600 | collagen, type I, alpha 1 | Homo sapiens |
| 806167 | serine/threonine kinase 36, fused homolog (Drosophila) | Homo sapiens |
| 798174 | cyclin A1 | Homo sapiens |
| 797559 | cyclin-dependent kinase 4 | Homo sapiens |
| 819591 | collagen, type IV, alpha 4 | Homo sapiens |
| 799249 | laminin, alpha 1 | Homo sapiens |
| 781132 | catenin (cadherin-associated protein), beta 1, 88kDa | Homo sapiens |
| 781978 | collagen, type VI, alpha 2 | Homo sapiens |
| 814431 | ras homolog gene family, member A | Homo sapiens |
| 781362 | cyclin-dependent kinase inhibitor 2A (melanoma, p16, inhibits CDK4) | Homo sapiens |
| 810626 | collagen, type V, alpha 3 | Homo sapiens |
| 796732 | v-akt murine thymoma viral oncogene homolog 2 | Homo sapiens |
| 798126 | matrix metallopeptidase 2 (gelatinase A, 72kDa gelatinase, 72kDa type IV collagenase) | Homo sapiens |
| 796239 | neuroblastoma RAS viral (v-ras) oncogene homolog | Homo sapiens |
| 819639 | death-associated protein kinase 1 | Homo sapiens |
| 823371 | laminin, gamma 1 (formerly LAMB2) | Homo sapiens |
| 810015 | prostaglandin-endoperoxide synthase 2 (prostaglandin G/H synthase and cyclooxygenase) | Homo sapiens |
| 818089 | v-rel reticuloendotheliosis viral oncogene homolog A (avian) | Homo sapiens |
| 804749 | wingless-type MMTV integration site family, member 5B | Homo sapiens |
| 786559 | son of sevenless homolog 2 (Drosophila) | Homo sapiens |
| 783742 | signal transducer and activator of transcription 5A | Homo sapiens |
| 822774 | laminin, beta 3 | Homo sapiens |
| 794222 | Ras association (RalGDS/AF-6) domain family member 5 | Homo sapiens |
| 781709 | vascular endothelial growth factor A | Homo sapiens |
| 798079 | cyclin-dependent kinase 6 | Homo sapiens |
| 796706 | v-raf murine sarcoma 3611 viral oncogene homolog | Homo sapiens |
